# Two Genetic Determinants Acquired Late in *Mus* Evolution Regulate the Inclusion of Exon 5, which Alters Mouse APOBEC3 Translation Efficiency

**DOI:** 10.1371/journal.ppat.1002478

**Published:** 2012-01-19

**Authors:** Jun Li, Yoshiyuki Hakata, Eri Takeda, Qingping Liu, Yasumasa Iwatani, Christine A. Kozak, Masaaki Miyazawa

**Affiliations:** 1 Department of Immunology, Kinki University School of Medicine, Osaka, Japan; 2 Laboratory of Molecular Microbiology, National Institute of Allergy and Infectious Diseases, National Institutes of Health, Bethesda, Maryland, United States of America; 3 Department of Infection and Immunology, Clinical Research Center, Nagoya Medical Center, Nagoya, Japan; University of Texas at Austin, United States of America

## Abstract

Mouse apolipoprotein B mRNA-editing enzyme catalytic polypeptide-like editing complex 3 (mA3), an intracellular antiviral factor, has 2 allelic variations that are linked with different susceptibilities to beta- and gammaretrovirus infections among various mouse strains. In virus-resistant C57BL/6 (B6) mice, mA3 transcripts are more abundant than those in susceptible BALB/c mice both in the spleen and bone marrow. These strains of mice also express mA3 transcripts with different splicing patterns: B6 mice preferentially express exon 5-deficient (Δ5) mA3 mRNA, while BALB/c mice produce exon 5-containing full-length mA3 mRNA as the major transcript. Although the protein product of the Δ5 mRNA exerts stronger antiretroviral activities than the full-length protein, how exon 5 affects mA3 antiviral activity, as well as the genetic mechanisms regulating exon 5 inclusion into the mA3 transcripts, remains largely uncharacterized. Here we show that mA3 exon 5 is indeed a functional element that influences protein synthesis at a post-transcriptional level. We further employed *in vitro* splicing assays using genomic DNA clones to identify two critical polymorphisms affecting the inclusion of exon 5 into mA3 transcripts: the number of TCCT repeats upstream of exon 5 and the single nucleotide polymorphism within exon 5 located 12 bases upstream of the exon 5/intron 5 boundary. Distribution of the above polymorphisms among different *Mus* species indicates that the inclusion of exon 5 into mA3 mRNA is a relatively recent event in the evolution of mice. The widespread geographic distribution of this exon 5-including genetic variant suggests that in some *Mus* populations the cost of maintaining an effective but mutagenic enzyme may outweigh its antiviral function.

## Author Summary

Susceptibility to acutely leukemogenic Friend virus (FV) retrovirus infection varies among different mouse strains and is governed by several genetic factors, one of which is allelic variations at the mouse *Apobec3* locus. FV-resistant C57BL/6 (B6) mice express higher amounts of *Apobec3* transcripts than susceptible BALB/c mice. We previously showed that the differences in N-terminal amino acid sequences between B6 and BALB/c APOBEC3 proteins partly account for the distinct antiretroviral activities. In addition, B6 and BALB/c mice express major *Apobec3* transcripts of different sizes: the exon 5-lacking and the full-length transcripts, respectively. Here we asked if exon 5 has any role in the antiviral activity of mouse APOBEC3 and found that the presence of this exon resulted in a profound decrease in the efficiency of protein synthesis without affecting the mRNA expression levels. We also identified two genomic polymorphisms that control the inclusion of exon 5 into the *Apobec3* message: the number of TCCT repeats in intron 4 and a single nucleotide polymorphism within exon 5. The distribution of these functional polymorphisms among *Mus* species and wild mouse populations indicates that the exon 5 inclusion occurred recently in *Mus* evolution, and the full-length variant may have selective advantages in some mouse populations.

## Introduction

The family of apolipoprotein B mRNA-editing enzyme catalytic polypeptide-like editing complex 3 (APOBEC3) proteins consists of cytidine deaminases that function as cellular restriction factors against various exogenous and endogenous viruses [1]–[17]. Seven APOBEC3 paralogues have been identified on human chromosome 22, while only a single copy of the *Apobec3* gene is found in the mouse genome [10], [18], [19]. Among the human APOBEC3 enzymes, APOBEC3G (hA3G) is the best characterized member and is known to inhibit HIV-1 replication when the virus lacks the functional accessory protein, viral infectivity factor (Vif) [reviewed in 20]. In the absence of Vif, hA3G is incorporated into newly generated virions budding from virus-producing cells and exhibits its antiviral effect in subsequently infected cells. Thus, during reverse transcription in the target cells, the virion-incorporated hA3G catalyzes C-to-U deamination on the minus strand of nascent viral DNA, resulting in G-to-A mutations on the plus strand of the double-stranded viral DNA, which can be detrimental to viral replication [7], [9], [10], [21], [22]. In addition, a deaminase-independent antiviral mechanism exerted by hA3G has also been reported [23], [24].

In contrast to its human counterparts, mouse APOBEC3 (mA3) restricts HIV-1 regardless of the presence of Vif, as well as mouse mammary tumor virus (MMTV), ecotropic murine leukemia viruses (MuLVs), Friend MuLV (F-MuLV) and Moloney MuLV (M-MuLV), along with endogenous mouse retroviruses including the AKR ecotropic virus (AKV) [5], [25]–[30]. This suggests that APOBEC3 enzymes protect host genomes from the retroviruses they commonly encounter, although some retroviruses, like HIV-1, have evolved to counter the intracellular restriction mechanisms of their natural hosts.

Friend virus (FV) is an acutely leukemogenic retroviral complex composed of replication-competent F-MuLV and replication-defective spleen focus-forming virus (SFFV). Susceptibilities to FV-induced disease development differ among various inbred strains of mice, and these are controlled by several host factors that either directly affect FV replication or influence host immune responses to the viral antigens [31], [32]. We and others have reported that the mouse *Apobec3* locus is polymorphic, and its genotypes are associated with the levels of viremia after F-MuLV or FV inoculation [26], [27]. Mice of the prototypic FV-resistant strains C57BL/6 (B6) and C57BL/10 exhibit restricted replication of F-MuLV and earlier production of FV-neutralizing antibodies, while FV-susceptible BALB/c and A strains are less restrictive of F-MuLV replication and show delayed production of neutralizing antibodies, all of which are linked with *Apobec3* genotypes [26], [27], [33], [34], although the production of neutralizing antibodies is also influenced by genotypes at the major histocompatibility complex and the *Tnfrsf13c* loci, the latter of which encode the receptor for B-cell activating factor belonging to the tumor necrosis factor family [31]–[33], [35].

Mouse APOBEC3 and hA3G contain two cytidine deaminase domains (CDDs), each harboring the conserved zinc-coordinating motif; however, deaminase activity is exerted only by the N-terminal CDD of mA3 and the C-terminal CDD of hA3G [36]. We showed that the increased efficiency of B6 mA3 in inhibiting F-MuLV replication is associated with differences in the primary amino acid sequence within the active N-terminal CDD [27]; the functional importance of these residues was further implicated by the demonstration that they have been under positive selection in *Mus* [37]. In addition to the above sequence differences in the protein-coding regions, efficient virus restriction is also associated with higher levels of mA3 transcripts in FV-resistant B6 than in -susceptible BALB/c mice [27], [29], [38], [39]. This enhanced transcription was linked with the presence of the long terminal repeat (LTR) of an endogenized xenotropic MuLV in the B6, but not in the BALB/c, *Apobec3* locus [37]. A third factor associated with virus resistance is the presence or absence of exon 5, which encodes a 33-amino acid segment separating the C-terminal and N-terminal CDDs [27]. The mA3 isoform of the FV-resistant strains of mice lacks exon 5, while the predominant transcript in FV-susceptible mice is the exon 5-containing isoform [27], [29], [37]. Genetic sequences controlling mA3 splicing have not been identified, although it has been pointed out that polymorphisms between the B6 and BALB/c *Apobec3* alleles at the end of intron 4 include a putative splice acceptor site and possible mRNA branch selection site structures [38]. It has also not been shown whether the well-confirmed differences in transcript levels result in altered expression levels of mA3 protein in FV-resistant and -susceptible strains of mice.

In the present report, we show that mA3 protein is indeed more abundant in B6 than in BALB/c mice. This difference is due in part to more efficient translation of the exon 5-deficient message. We further show extensive functional evidence that two distinct polymorphisms within the *Apobec3* locus regulate exon 5 inclusion during its splicing: the previously predicted [38] TCCT repeat numbers in intron 4 and a newly identified single nucleotide polymorphism (SNP) within exon 5. We also describe the linkage between these splicing regulatory sequences in wild mouse species, their acquisition in *Mus* evolution, and their distribution in wild mouse populations.

## Results

### APOBEC3 expression is higher in B6 than in BALB/c spleens at both transcriptional and protein levels

**Figure 1:**
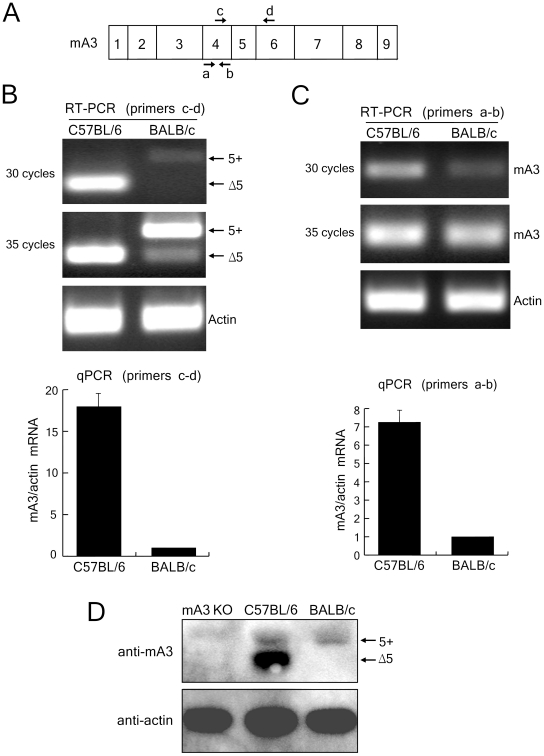
Expression of mA3 transcripts and protein in the spleens of B6 and BALB/c mice. (A) Schematic representation of the mouse APOBEC3 exon composition. The exons are indicated by numbers and their relative sizes are depicted. The arrows indicate the locations of the PCR primers used in the following experiments. (B and C) RT-RCR and quantitative real-time PCR (qPCR) analyses showing splicing patterns and quantities of mA3 mRNA in B6 and BALB/c spleen cells. The primers utilized were c-d for assays shown in (B) and a–b for those in (C). 5+, exon 5-containing mA3; Δ5, mA3 lacking exon 5. Actin was used as an internal control. Quantitative real-time PCR data show averages of three reaction wells and SD. (D) Immunoblot detection of mA3 protein expressed in mouse spleen cells. The spleen cell extracts were tested for their total protein content by Bradford assays and the same amount of protein was loaded in each lane. Anti-mouse APOBEC3 NT antibody reactive with an N-terminal epitope was first pre-absorbed with the spleen lysate prepared from an mA3-knockout (KO) mouse, and was used as the primary antibody. Mouse actin was used as a loading control. Three independent experiments were done for all assays, and the results were consistent with the representative ones shown here.

It has been reported that mA3 mRNA expression is higher in B6 than in BALB/c mice, and the mA3 transcripts detected in B6 mice are predominantly the exon 5-lacking Δ5 isoform, while the majority of mA3 transcripts in BALB/c mice contain exon 5 (5+) [27], [29], [38]. To more accurately describe the above quantitative differences in mA3 transcripts, we performed two types of PCR analyses with two different sets of specific primers (Figure 1A). The primer set c–d, which is the same as the one previously used [27], detected both the 5+ and Δ5 transcripts in reverse-transcription PCR (RT-PCR) assays; however, mA3 transcripts were barely detectable after 30 cycles of amplification in BALB/c mice while the Δ5 transcript was readily detectable in B6 mice (Figure 1B). After 35 cycles of amplification, both the 5+ and Δ5 transcripts became detectable in BALB/c mice, although the 5+ mRNA was more abundant. Quantitative real-time PCR (qPCR) assays revealed mA3 expression levels that were approximately 18-times higher in B6 than in BALB/c spleens, although the difference in transcript lengths might have influenced the efficiencies of amplification in the real-time PCR reactions. To more precisely quantify the mA3 transcripts, we utilized the second primer set, a–b, which generates amplicons of the same size from both alleles (Figure 1A). The qPCR assay performed by using this latter primer set clearly demonstrated that B6 mice expressed >7-times higher amounts of mA3 transcript than BALB/c mice did (Figure 1C), consistent with the previous report [27]. The normalization of mA3 mRNA levels with TATA box-binding protein or GAPDH transcripts instead of β-actin gave similar results (data not shown).

Protein levels of mA3 in the above prototypic FV-resistant and -susceptible strains of mice were also compared. The spleen lysate from the mA3-knockout mice [40] was used as a negative control. In B6 spleens, an immunoreactive protein corresponding to Δ5 mA3 was detected as a prominent band, but the higher molecular weight 5+ mA3 was also faintly detectable (Figure 1D), even though the 5+ message was barely detectable by RT-PCR in the present study (Figure 1B) and in the previous reports [27], [29]. On the other hand, only the 5+ mA3 protein was detected, with a much lower intensity, in BALB/c spleens. The immunoblotting assays thus demonstrated that the level of total mA3 protein expression in the spleens of B6 mice is much higher than that in BALB/c mice.

### Effects of exon 5 inclusion on mA3 protein expression

**Figure 2:**
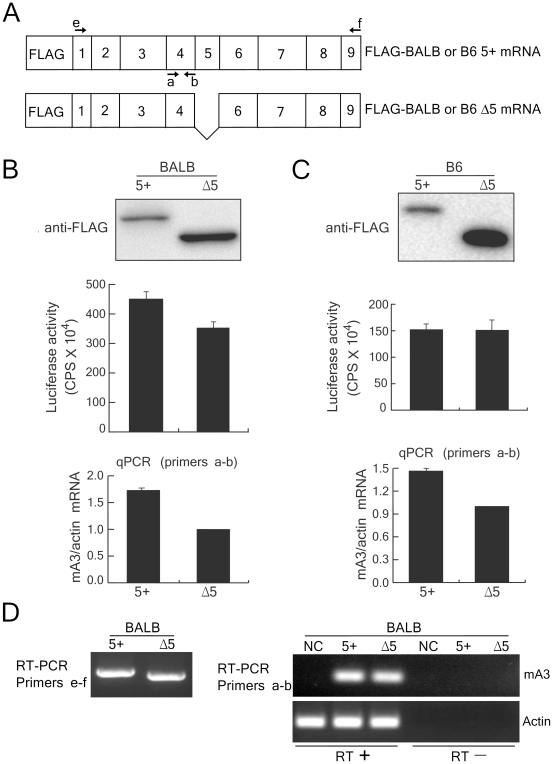
Protein expression of the full-length and Δ5 mA3 in transiently transfected 293T cells. (A) Exon 5-containing (5+) and Δ5 mA3 cDNA were tagged with the FLAG epitope and inserted into the expression vector. The arrows indicate the positions of the PCR primers. The primer set a-b is the same as shown in Figure 1. (B and C) 293T cells were transfected with pFLAG-CMV2-*mA3^d^* or pFLAG-CMV2-*mA3^d^*Δ5 in B or with pFLAG-CMV2-*mA3^b^* or pFLAG-CMV2-*mA3^b^*Δ5 in C, which express either the 5+ or Δ5 mA3 cDNA cloned from BALB/c or B6 mice, respectively [27]. A luciferase-expressing plasmid, p*luc*, was co-transfected to standardize transfection efficiencies. At 24 hours after transfection, each one-third of the transfected cells was used for immunoblotting, RNA extraction, and luciferase assays. For immunoblotting, the full-length and Δ5 mA3 proteins were detected with the anti-FLAG antibody. Quantitative real-time PCR reactions were carried out with primer set a-b and were normalized with the levels of actin transcripts expressed in 293T cells. Data shown are averages of three reaction wells and SD. (D) RT-PCR assays were performed with (RT+) or without (RT−) reverse transcription to demonstrate specific detection of expected mRNA. Both primer sets e-f and a-b were used to detect mA3 mRNA. NC, negative control transfected with the empty vector, pFLAG-CMV2. RT– samples were included to evaluate the possible contamination of transfected DNA. Specific amplification of cDNA generated in the presence of RT was observed in the cells transfected with a plasmid expressing the 5+ or Δ5 mA3 cDNA. Similar results were obtained for the corresponding B6-derived cDNA clones.

The mA3 expression data shown in Figure 1 indicate that levels of mA3 protein expression roughly correlate with the corresponding transcript levels in mice with different *Apobec3* alleles. However, we previously observed that even when FLAG-tagged mA3 was expressed under the control of the cytomegalovirus (CMV) promoter, the levels of expression of the 5+ mA3 protein tended to be much lower than those of the Δ5 protein, despite similar levels of mRNA expression in transfected cells [27]. This implies that exon 5 might affect either the efficiency of mA3 translation or protein stability. To evaluate these possibilities, 5+ and Δ5 mA3 expression plasmids were constructed with either B6- or BALB/c-derived mA3 cDNA (Figure 2A). The mA3 cDNA were fused with a FLAG tag at their N-terminus and the transcription was driven by the CMV promoter. After transfection of 293T cells with either one of the FLAG-tagged expression constructs, we quantified mA3 transcripts by qPCR and mA3 protein by immunoblotting (Figure 2, B–D). Based on the co-expressed luciferase activities, similar transfection efficiencies for the 5+ and Δ5 plasmids were confirmed, and the data from the qPCR showed that the 5+ mA3 transcript was more highly expressed than the Δ5 mRNA for both alleles. Nevertheless, the expression levels of the 5+ mA3 protein were lower compared to those of the Δ5 counterpart regardless of the allelic differences, although B6 Δ5 protein was expressed more abundantly than BALB/c Δ5 (Figure 2, B and C). These results indicate that the inclusion of exon 5 does not compromise mA3 mRNA expression, but decreases the steady-state levels of mA3 proteins for both allelic variants.

### Exon 5 inclusion does not affect mA3 protein stability but influences its translation

**Figure 3:**
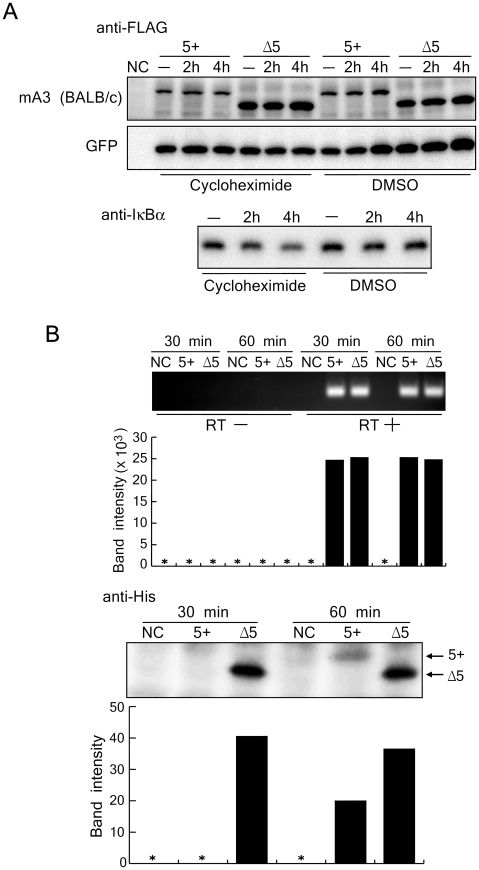
The presence of exon 5 does not affect protein degradation but exhibits a profound impact on mA3 protein synthesis. (A) 293T cells were transfected with pFLAG-CMV2-*mA3^d^* or pFLAG-CMV2-*mA3^d^*Δ5 along with pFLAG-CMV2-GFP, which expresses green fluorescent protein (GFP) as a loading control. The cells were treated with cycloheximide for the indicated duration to stop protein synthesis and then harvested. DMSO was used as a solvent control. The FLAG-tagged mA3 and GFP were detected with the anti-FLAG antibody in immunoblotting. Endogenous IκBα expression was used as a positive control to confirm the effect of cycloheximide. (B) *In vitro* transcription and translation assays. DNA templates containing the entire coding region of the BALB/c full-length or Δ5 mA3 cDNA with the T7 promoter and His-tag sequence were subjected to an *in vitro* transcription/translation reaction by incubating for 30 or 60 min. The levels of mA3 protein synthesis and mRNA expression were evaluated by immunoblotting using the anti-His antibody and RT-PCR using the primer set a-b, respectively. Intensities of protein bands on the immunoblot membrane and DNA bands after the RT-PCR reaction and electrophoresis were measured by densitometry and are shown below each corresponding band. *, signals below detection limits.

As higher mRNA levels of the 5+ mA3 resulted in lower protein levels in comparison with those for the Δ5 isoform in transfected cells, we next examined the steps at which the synthesis or degradation of mA3 protein was affected by the presence of exon 5. To this end, we transfected 293T cells with an expression plasmid harboring the 5+ or Δ5 mA3 cDNA, and cycloheximide was added to stop protein synthesis. The inhibitor of nuclear factor-κB, IκBα, was utilized as a control, as this protein is known to be in the process of constant degradation and regeneration under serum-containing culture conditions [41]. As expected, immunodetectable IκBα decreased upon cycloheximide treatment, while the solvent alone did not affect the protein content (Figure 3A). On the other hand, mA3 expressed in the same cells did not exhibit any reduction upon cycloheximide treatment regardless of the presence or absence of exon 5. Further, the Δ5 protein was again detected at higher levels than the 5+ protein. These results support the conclusion that the effect of exon 5 inclusion in reducing the amount of expressed mA3 protein is not due to accelerated protein degradation.

We next utilized an *in vitro* transcription and translation procedure to determine if exon 5 affects the translation of mA3 (Figure 3B). When the 5+ or Δ5 mA3 templates were added to the *in vitro* transcription and translation reaction, similar amounts of each transcript were detected by RT-PCR assays both at 30 min and 60 min after the beginning of incubation. However, the tempos of appearance and amounts of the 5+ mA3 protein were different from those of the Δ5 counterpart: a large amount of the Δ5 protein was detected at as early as 30 min after the beginning of incubation, while the 5+ mA3 was undetectable at the same time-point. The 5+ mA3 protein became detectable after 60 min of incubation, but its protein level was still markedly lower than that of the Δ5 counterpart. These results collectively indicate that the inclusion of exon 5 modulates the translation efficiency of mA3 rather than its protein degradation.

### The TCCT repeat number variation upstream of exon 5 is partially responsible for exon 5 inclusion into the mA3 transcript

**Figure 4:**
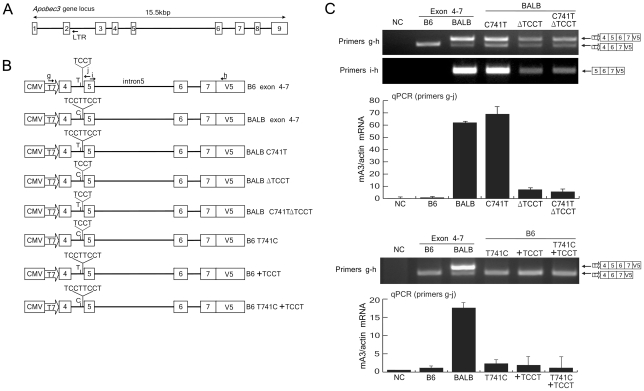
The impact of TCCT repeat numbers on exon 5 inclusion in mA3 mRNA splicing. (A) Schematic representation of the genomic DNA structure of the *Apobec3* gene locus. Boxes indicate exons. LTR, the endogenous retroviral LTR inserted into intron 2 in some strains of mice [37]. (B) Insert structures of the plasmids used for the splicing assays. BALB/c exon 4–7 and BALB/c ΔTCCT plasmids harbored the same DNA fragment amplified from BALB/c genomic DNA encompassing exons 4 and 7, except that the BALB/c ΔTCCT contains only a single TCCT quadruplet. B6 exon 4–7 plasmid harbored the B6 genomic DNA fragment encompassing exons 4 and 7. C741T and T741C indicate a C to T or reciprocal nucleotide substitution, respectively, within intron 4 at 741-bp downstream from the first nucleotide of exon 4 on the backbone of the BALB/c or B6 exon 4–7 insert. An additional repeat of the TCCT quadruplet was added to the B6 exon 4–6 construct to generate B6 +TCCT. The positions of primers g, h, i, and j used for RT-PCR assays are indicated with the arrows. (C) The plasmid harboring each insert depicted in (B) was transfected into BALB/3T3 cells, total RNA was extracted, and RT-PCR reactions were performed with primer pairs g–h and i–h. A portion of the transfected cells were utilized for luciferase assays to compare transfection efficiencies among the samples. Comparable levels of luciferase activities were observed in all samples in each experiment (data not shown). The predicted splicing products are schematically indicated on the right side of the panel. Quantitative real-time PCR assays were performed with primers g and j, and data are shown here by averages of three reaction wells and SD.

As the inclusion of exon 5 is associated with a reduced level of mA3 protein, we next attempted to identify genetic polymorphisms that affect the splicing patterns of mA3 transcripts in terms of exon 5 inclusion. One possible allelic difference in the mouse *Apobec3* locus putatively associated with its splicing patterns is the possible pre-mRNA branch site polymorphisms found in the intron upstream of exon 5: a T/C SNP that lies within a preferred branch site sequence, TA(T/C)CAAC, and TCCT repeat numbers between this and the acceptor site [38]. As described previously [38], the intron 4 of the BALB/c allele contains a tandem repeat of the TCCT sequence near the intron 4/exon 5 boundary, while the B6 allele contains only a single TCCT copy, changing the length of the putative pyrimidine-rich lariat intermediate. The adjacent T/C SNP at position 741 from the first nucleotide of exon 4 is in linkage with the TCCT repeat number. To directly investigate the possible effect of these polymorphisms, we constructed splicing assay plasmids that harbor the *Apobec3* genomic fragment encompassing exons 4 through 7 from either the B6 or BALB/c allele. The resultant plasmids were designated B6 exon 4–7 and BALB exon 4–7 (Figure 4). Three mutants of each of these constructs were made with different combinations of the position 741 T/C SNP and the TCCT copy number variation as depicted in Figure 4B.

Because of the possible presence of species-specific regulatory factors, the resultant genomic constructs were transfected into BALB/3T3 instead of 293T cells along with the luciferase expression plasmid as a control for transfection efficiency. To avoid the amplification of the endogenous mA3 message expressed in BALB/3T3 cells [27], we utilized the primers g and h (Figure 4B) for RT-PCR assays, which were designed to hybridize to the T7 promoter and V5 tag regions of the expression vector. Transfection with the B6 exon 4–7 plasmid resulted in the expression of only the Δ5 transcript, while the BALB exon 4–7 generated both the 5+ and Δ5 transcripts with much higher intensity of the 5+ one (Figure 4C), reproducing the splicing patterns observed in B6 and BALB/c spleens, respectively (Figure 1B). The ratio between the 5+ and Δ5 transcripts was reduced in the samples transfected with BALB ΔTCCT or BALB C741T ΔTCCT plasmid harboring only a single copy of TCCT, which was further confirmed by utilizing the primer i hybridizing to the sequence within exon 5 along with primer h (Figure 4C). Quantitative real-time PCR analyses were done by utilizing another primer (primer j), designed to hybridize with the sequence within exon 5, and further confirmed reduced levels of the exon 5-containing message expressed from the modified BALB exon 4–7 constructs lacking the TCCT repeat. These results imply that the TCCT repeat observed in the BALB/c allele, but not the T/C substitution at position 741, at least partly facilitates the exon 5 inclusion. The lack of an effect of the T/C SNP within the putative branch site sequence was further confirmed by the abundant expression of the 5+ message from the BALB C741T construct. However, both the 5+ and Δ5 transcripts were still produced from the BALB ΔTCCT plasmid, suggesting that the TCCT repeat number is not the only determinant controlling the exon 5 inclusion. In fact, despite the presence of the repeated TCCT, B6 +TCCT and B6 T741C +TCCT did not generate the 5+ message, while transfection with the corresponding BALB C741T and the wild-type BALB exon 4–7 clearly resulted in the generation of the 5+ mRNA. These results indicate that polymorphisms other than those in intron 4 might play more important roles in determining exon 5 inclusion into mA3 messages.

### A region around the exon 5/intron 5 boundary contains a major determinant for exon 5 inclusion

**Figure 5 ppat-1002478-g005:**
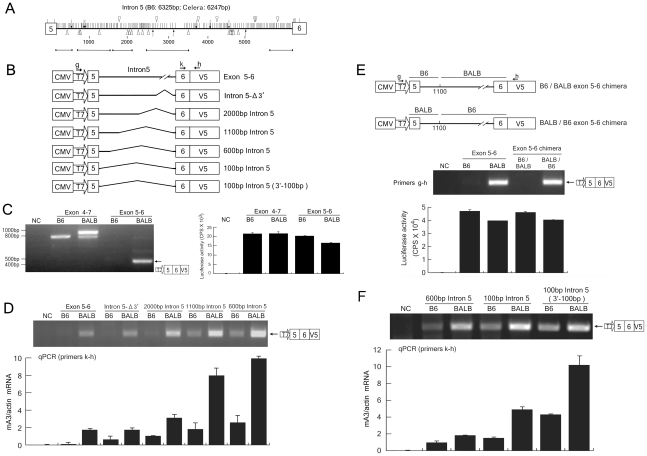
The effect of exon 5 and its downstream sequences on mA3 intron 5 splicing. (A) Distributions of sequence polymorphisms in the intron 5 of the *Apobec3* gene locus between the B6 allele [NT_039621] and the Celera database sequence of mixed mouse DNA [NW_001030577.1]. Shorter horizontal lines below the thick one representing intron 5 indicate regions of sequenced BALB/c genome [DDBJ accession No. AB646261-AB646265], which show nucleotide sequences identical to corresponding Celera database sequences. Spans of the analyzed regions are 1–620, 661–1597, 1643–2058, 2759–3443, and 5558–6247 in base numbers starting from the first nucleotide of intron 5. SNP are shown with vertical lines, single-base indels with arrows, and deletions of ≥2 bases with triangles. Indels and deletions above the thick horizontal line that represents intron 5 are deletions in the B6 allele relative to the Celera sequence, while those underneath the horizontal line are deletions in the Celera sequence relative to the B6 allele sequence. (B) Plasmid constructions for the splicing assays. The exon 5–6 plasmids harbored either the B6 or BALB/c genomic fragment encompassing exons 5 and 6, including the entire intron 5 of the corresponding allele. Each of the remaining plasmids possessed a sequentially reduced length of the intron 5 as indicated. The precise size of each PCR-generated 5′ fragment included was as follows: 3177bp and 3185bp for B6 and BALB/c intron 5-Δ3′; 2106bp and 2086bp for B6 and BALB/c 200bp intron 5; 1120bp and 1110bp for B6 and BALB/c 1100bp intron 5; and 634bp and 620bp for B6 and BALB/c 600bp intron 5, respectively. The primers g–h are the same as shown in Figure 4. (C) RT-PCR detection of spliced messages expressed from the B6 and BALB/c exon 5–6 plasmids along with those from the exon 4–7 plasmids as controls. Primers g and h were used. A lack of expression of the spliced message in cells transfected with the B6 exon 5–6 plasmid was evident. (D and F) Splicing assays using intron 5 deletion plasmids. The intron 5 fragment included in each plasmid is shown in (B). RT-PCR assays were performed with primers g and h. A portion of the transfected cells were utilized for luciferase assays to compare transfection efficiencies among the samples as shown in (C). Comparable levels of luciferase activities were observed in all samples in each experiment (data not shown). Quantitative real-time PCR data show averages of three reaction wells and SD. (E) Reciprocal chimeras were produced between B6 and BALB/c exon 5–6 by exchanging the cloned genomic DNA fragment at position 1100 within intron 5. The exact location of the above position 1100 for B6 and BALB/c intron 5 is described in the legend for (B). RT-PCR detection of spliced messages was performed with primers g and h. A portion of the transfected cells were utilized for luciferase assays to compare transfection efficiencies.

As abundant expression of the exon 5-containing message was found with the BALB C741T but not with the B6 +TCCT construct, we were prompted to examine other polymorphisms downstream of intron 4 that might be involved in the splicing of exon 5. To explore other possible sequence variations required for exon 5 inclusion, we focused on intron 5, since this region contains numerous polymorphisms between reported genomic sequences of different mouse strains (Figure 5A). Splicing assay vectors harboring the genomic DNA fragment containing exons 5 and 6 along with the entire intron 5, from either the B6 or the BALB/c allele, were constructed and designated B6 exon 5–6 or BALB exon 5–6 (Figure 5B). BALB/3T3 cells transfected with the BALB exon 5–6 plasmid generated the properly spliced product of the expected size (Figure 5C), indicating that the splice donor site downstream of exon 5 and the acceptor site upstream of exon 6 in the BALB/c allele are both functional. In contrast, no spliced product was detectable in cells transfected with the B6 exon 5–6 plasmid despite a higher transfection efficiency. These results indicate that the B6 allele may not carry a functional splice donor site at the exon 5/intron 5 boundary, as the splice acceptor site upstream of exon 6 seems intact and thus can generate a message corresponding to the mA3 Δ5 when exon 4 and intron 4 are included (Figures 4 and 5C).

To narrow down the region affecting the intron 5 splicing, serial deletion constructs were produced and subjected to the splicing assay (Figure 5B). The intron 5-Δ3′ is a deletion construct which lacks the 3′ half of intron 5 but retains the 261-bp sequence adjacent to exon 6 to include the putative acceptor site. The cells transfected with the BALB intron 5-Δ3′ plasmid produced the spliced message as efficiently as those transfected with the parental BALB exon 5–6 construct; however, the B6 intron 5-Δ3′ plasmid generated a barely detectable band representing spliced message (Figure 5D). Quantitative real-time PCR assays revealed markedly lower levels of properly spliced message generated from the B6 intron 5-Δ3′ plasmid compared to that generated from the BALB/c counterpart. These data show that the 5′ half of intron 5 is responsible for the observed differences in splice site functions between the B6 and BALB/c alleles. Thus, further deletions progressively closer to exon 5 were introduced into the intron 5 fragment. As expected, the shorter the included intron 5 fragment, the more abundant the spliced product generated was, regardless of the expressed *Apobec3* alleles, indicating that the intron length of the primary transcript influences the splicing efficiency (Figure 5D). Nevertheless, the spliced product was generated much more efficiently from the BALB/c allele than from the B6 allele with all examined construct pairs, despite comparable levels of transfection efficiency, indicating that each shorter intron fragment still retained the polymorphism responsible for the inefficient splicing of the B6 mRNA.

To exclude the possibility that the 3′ region of intron 5 and/or exon 6 from the B6 allele might harbor inhibitory sequences that interfere with splicing, we constructed chimeras between the BALB and B6 exon 5–6 plasmids by exchanging the corresponding genomic DNA fragments at approximately position 1100 within intron 5 (Figure 5E). BALB/B6 chimera harboring the 5′ donor site sequence from the BALB/c allele and 3′ acceptor site from the B6 allele generated properly spliced mRNA as efficiently as the BALB exon 5–6 plasmid did, while the reciprocal construct harboring the B6 donor and BALB/c acceptor sequences did not (Figure 5E), indicating that there are no inhibitory elements in the fragment harboring the 3′ intron 5 and exon 6 from the B6 allele. Thus, we continued to narrow down the sequence affecting the intron 5 splicing toward the 5′ end of the genomic constructs.

Even the shortest construct pair, B6 100bp intron 5 and BALB 100bp intron 5, which harbor the 5′ intron 5 fragment a mere 100-bp from the exon 5 boundary, still exhibited a readily discernible difference in the amounts of the spliced messages, and this was also true for another pair of deletion constructs that harbor a 100-bp acceptor region fragment of 3′ intron 5 (Figure 5F). These results clearly indicate that the region including exon 5 and the 5′ 100-bp of intron 5 carries the primary determinants responsible for the different efficiencies in splicing of intron 5 shown by the B6 and BALB/c alleles.

### A single nucleotide polymorphism within exon 5 is primarily responsible for exon 5 inclusion

**Figure 6 ppat-1002478-g006:**
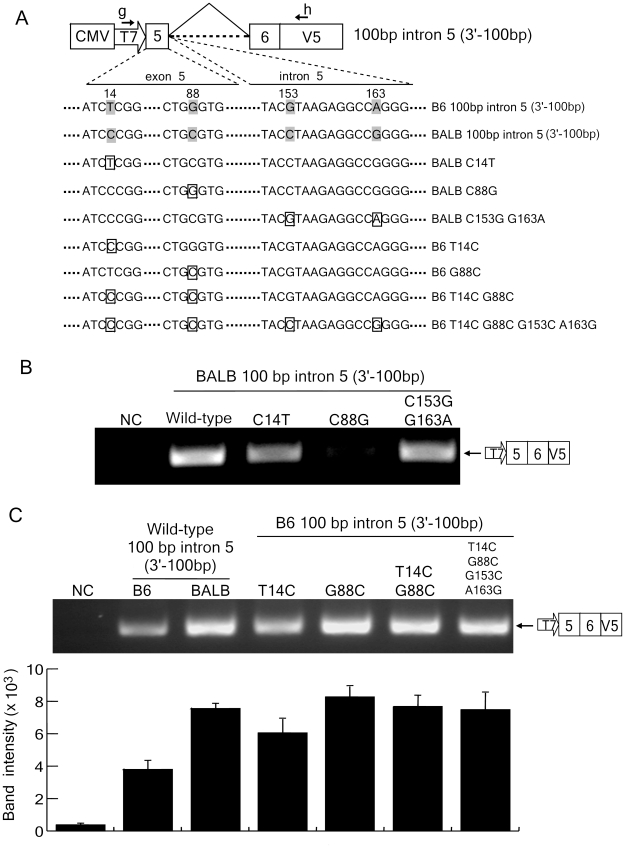
Identification of critical residues for exon 5 inclusion into mA3 mRNA. (A) Polymorphic nucleotides indicated with the shades were exchanged at the boxed sites between the B6 and BALB/c alleles. The numbers 14, 88, 153, and 163 above the shown nucleotide sequence are base numbers from the first nucleotide of exon 5. (B and C) The results of splicing assays performed with the plasmids depicted in (A). The predicted splicing product is shown on the right side of each panel. Band intensities are averages of 3 independent RT-PCR reactions with SD.

Within the above narrowed-down region of exon 5 and the 5′ 100 bp of intron 5, there are only 4 SNPs between the B6 and BALB/c alleles, as previously described [38]: the T/C SNP at 14-bp downstream from the first nucleotide of exon 5, G/C at 88-bp downstream from the same first nucleotide, G/C at 153-bp downstream within intron 5, and A/C at 163-bp downstream within intron 5 (Figure 6A). We therefore produced a series of point mutants based on the B6 intron 5–100 (3′-100bp) and BALB/c intron 5–100 (3′-100bp) constructs to precisely identify the critical SNP responsible for the functionalities of the splice donor site. In the first set of experiments, shown in Figure 6B, each indicated nucleotide within the BALB/c genomic sequence was substituted with the corresponding nucleotide found in the B6 allele. The substitution of the C at position 14 to the B6-type T slightly reduced the amount of the spliced message; more importantly, however, the similar substitution of C at position 88 to the B6-type G totally abrogated the intron 5 splicing. Simultaneous substitutions of C to G at position 153 and G to A at 163 did not affect the generation of the spliced message. Transfection efficiencies were essentially equivalent for all samples and even higher than those for the C88G construct as confirmed by cotransfecting the luciferase expression plasmid (data not shown). These results indicate that the G/C SNP at position 88 in exon 5 is most critical for the splice donor function.

In a reciprocal set of experiments, we attempted to alter splicing of the transcript of the B6 allele by replacing the polymorphic nucleotides with the BALB/c-types (Figure 6C). When all 4 SNPs in the B6 intron 5–100 fragment were replaced with those corresponding to the BALB/c allele, abundant generation of the spliced message was observed (Figure 6C). A combined substitution of T to C and G to C at positions 14 and 88, respectively, also resulted in efficient splicing of intron 5, and a single substitution of T to C at position 14 alone slightly increased the generation of the spliced message from the plasmid harboring the truncated intron. Importantly, a single substitution of G to C at position 88 resulted in as abundant expression of the spliced message as that observed with the BALB/c allele. Thus, these results collectively indicate that the G at position 88 is critical for the exclusion of exon 5, although the C at position 14 may also play a minor role in the inclusion of this exon.

### Site-directed mutagenesis of the plasmids harboring the entire intron 5

**Figure 7:**
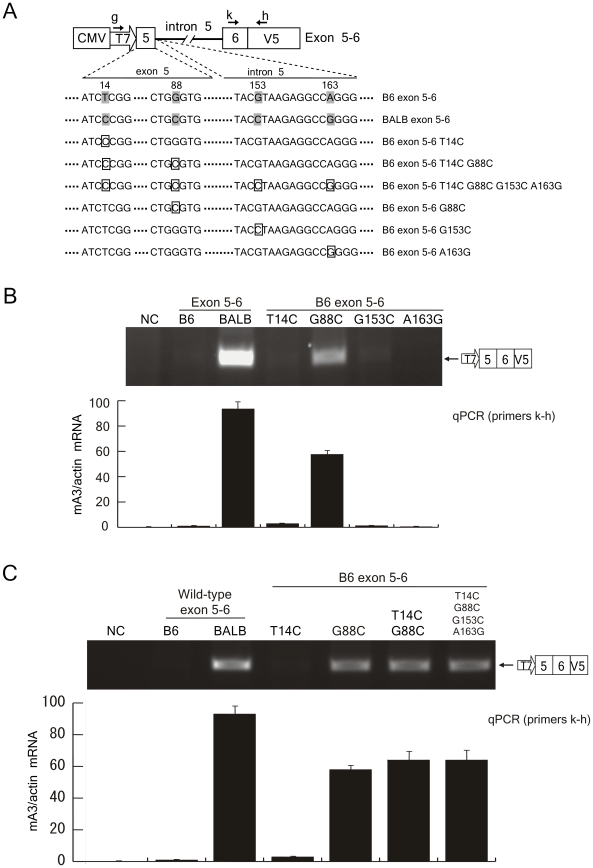
The G88C nucleotide substitution in exon 5 is most critical for exon 5 inclusion into mA3 mRNA. (A) Polymorphic nucleotides indicated with the shades were exchanged between the B6 and BALB/c alleles at the sites indicated with the boxes. Plasmids used here carry the entire intron 5 instead of the deleted one used in the experiments shown in Figure 6. (B and C) The results of splicing assays using the plasmids depicted in panel (A). The predicted splicing product is shown on the right side of each panel. RT-PCR assays were performed with primers g and h. A portion of the transfected cells were utilized for luciferase assays to compare transfection efficiencies among the samples. Comparable levels of luciferase activities were observed in all samples in each experiment (data not shown). Quantitative real-time PCR data show averages of three reaction wells and SD.

To determine the association of the above 4 SNPs with splicing efficiency under more physiological conditions, we further introduced point mutations into the B6 exon 5–6 plasmid harboring the entire intron 5 of 6kb in length, and the resultant plasmids were subjected to the splicing assays (Figure 7A). In contrast to the result with the B6 100bp intron 5 T14C mutant (Figure 6C), we could not detect the generation of spliced mRNA after transfection of the B6 exon 5–6 T14C mutant (Figure 7B). However, the single substitution at position 88 from the B6-type G to BALB/c-type C led to readily detectable production of the spliced mRNA, despite the general inefficiency in splicing of the full-length intron 5 depicted in Figure 5. Quantitative real-time PCR assays revealed levels of expression of the exon 5-containing spliced message from the B6 exon 5–6 G88C mutant that were about 70% of those expressed from the BALB exon 5–6 plasmid. Single nucleotide substitutions at two other positions did not result in a detectable generation of the spliced mRNA from the B6 allele (Figure 7B). Combined mutations at positions 14 and 88 within exon 5 on the B6 exon 5–6 construct (B6 exon 5–6 T14C G88C) and further addition of the nucleotide replacements at positions 153 and 163 also resulted in detectable generation of the spliced message, but did not noticeably enhance the splicing over what resulted from the G88C substitution alone (Figure 7C).

### Combined effects of the intron 4 TCCT repeat number and exon 5 G/C SNP on exon 5 inclusion into mA3 mRNA

**Figure 8:**
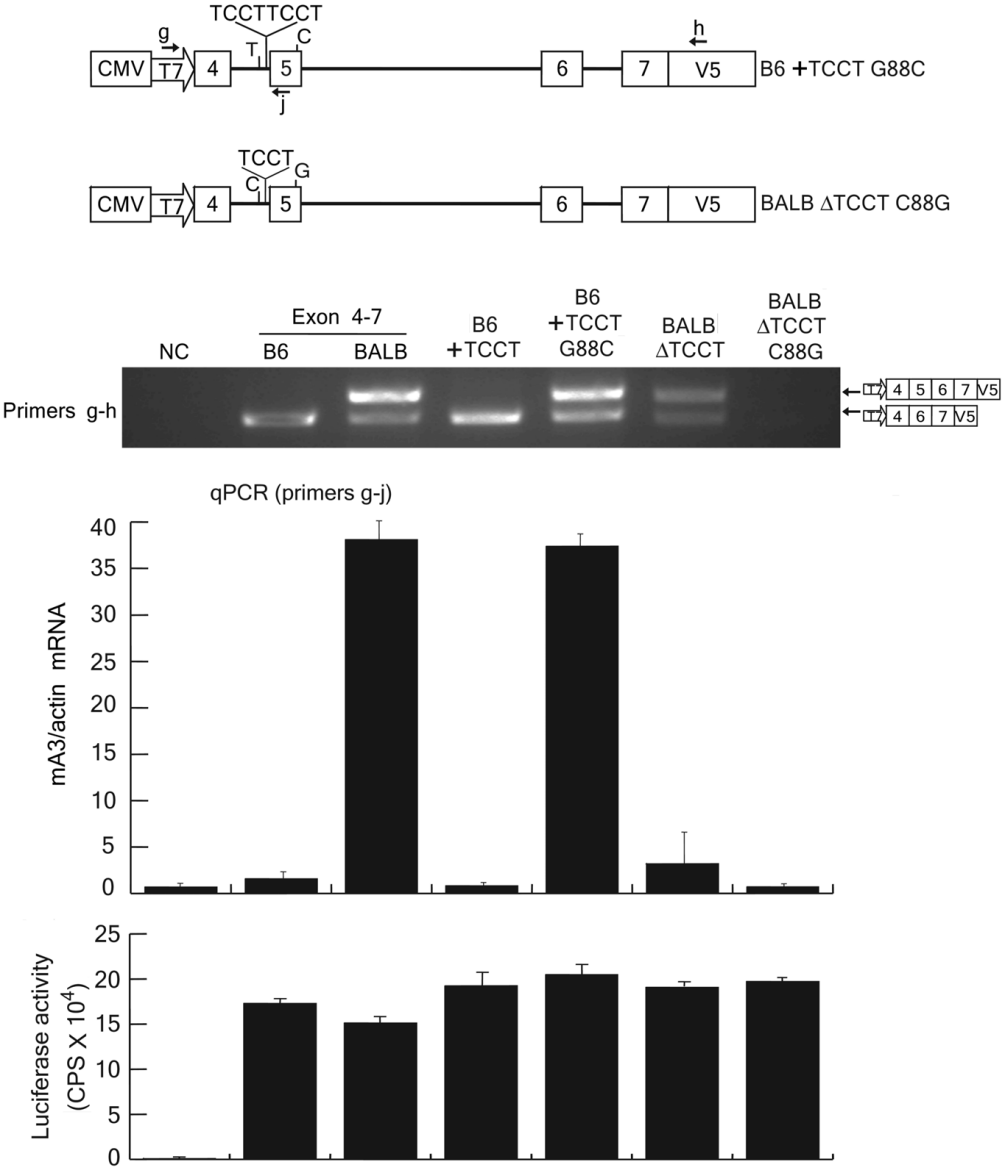
Effect of the combination of intron 4 TCCT repeat and exon 5 G/C SNP on exon 5 inclusion into the mA3 transcripts. B6 exon 4–7 + TCCT and BALB/c exon 4–7 ΔTCCT plasmids, as described in Figure 4, were further modified so that the exon 5 G/C SNP nucleotide at position 88 was reciprocally exchanged. The resultant plasmids were used for *in vitro* transcription assays as described for Figures 4–7. RT-PCR assays were performed with primers g and h. A portion of the transfected cells were utilized for luciferase assays to compare transfection efficiencies among the samples. Quantitative real-time PCR data show averages of three reaction wells and SD.

To further compare the effects of the TCCT repeat number and exon 5 G/C SNP at position 88, we constructed B6 and BALB exon 4–7 plasmids that harbored reciprocal substitutions at these two polymorphic sites (Figure 8). Introduction of an additional copy of TCCT into the B6 exon 4–7 construct did not result in the generation of 5+ message, while the combination of the additional TCCT and G88C substitution resulted in the generation of exon 5-containing message at levels comparable to those expressed from the BALB exon 4–7 plasmid. On the other hand, a deletion of a TCCT copy from the BALB exon 4–7 construct resulted in much reduced expression of the 5+ message, and the combination of ΔTCCT and C88G substitution totally abrogated the generation of exon 5-containing message. Thus, these results clearly demonstrate that the most critical polymorphism for exon 5 inclusion into the mA3 mRNA is the G/C SNP at position 88 within exon 5, but for the full-level expression of the 5+ mRNA as observed in BALB/c mice the intron 4 TCCT repeat is also required.

### Distribution of the TCCT repeat and G/C88 polymorphism among *Mus* species

**Figure 9:**
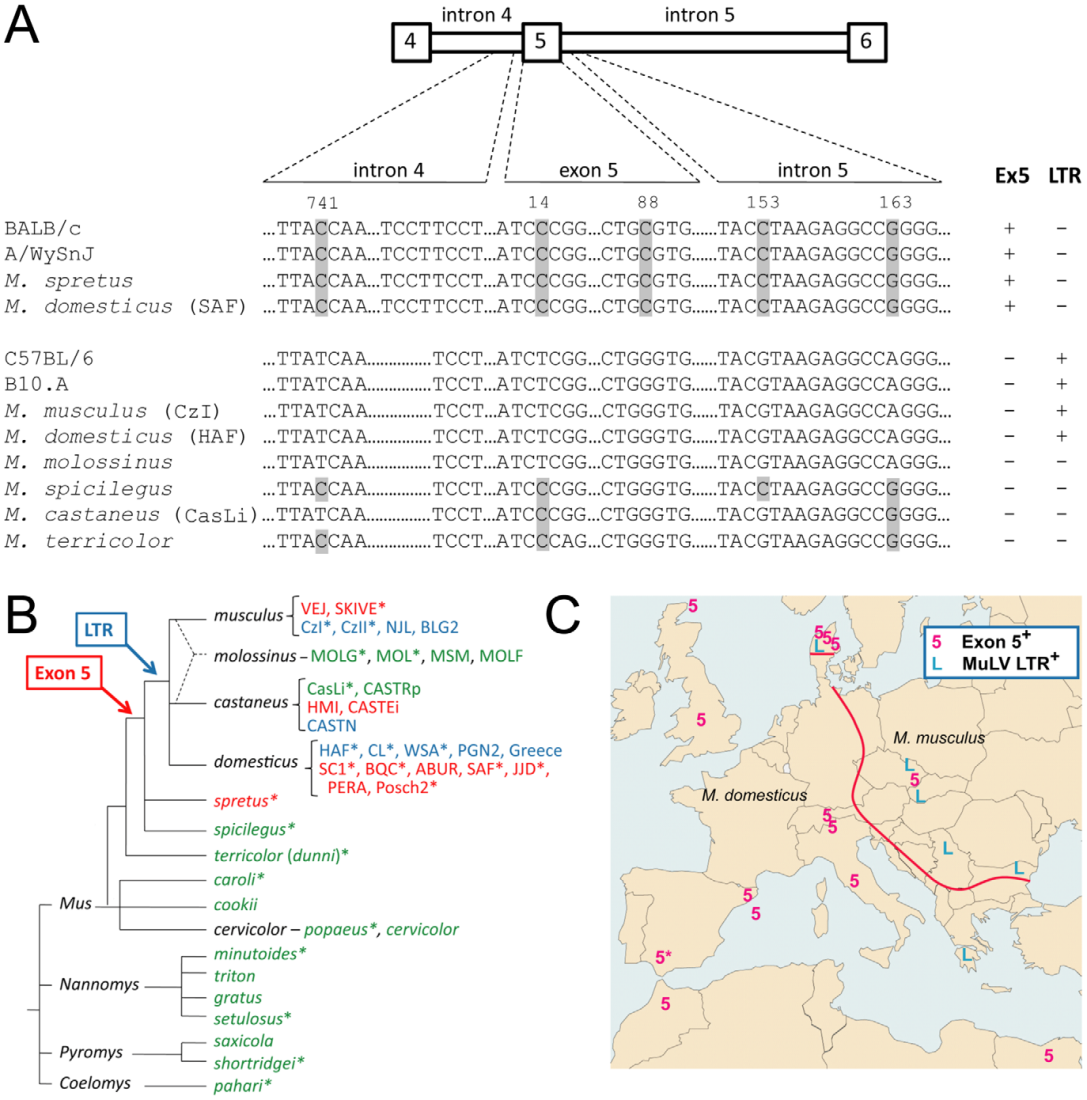
Distribution of sequence variations in and around the *Apobec3* exon 5 in *Mus* species and strains. (A) The *Apobec3* genomic sequences of the indicated strains and representative species are shown. The five SNP tested for their effects on exon 5 splicing are indicated with shaded bars. Also shown are the number of TCCT repeats and typing results indicating the presence or absence of exon 5 (Ex5) in spliced messages and the presence or absence of an MuLV LTR in intron 2. (B) The phylogenetic tree is a synthetic tree adapted from other sources [66]–[68]. Species are grouped into the 4 *Mus* subgenera and are listed to the right of each branch. Subspecies and stock or inbred line designations are given where multiple samples of a given species were tested. Red, exon 5^+^/MuLV LTR^−^; blue, Δ5/MuLV LTR^+^; green, Δ5/MuLV LTR^−^. *, mice tested for exon 5 splicing pattern. All were sequenced across exon 5 (Figure S1); PERA and MOLF sequence information was taken from Okeoma et al. [38]. (C) Distribution of expression variants of mA3 in mice trapped in Europe. The red line represents the 20 km-wide hybrid zone separating the ranges of *M. domesticus* and *M. musculus*. Symbols representing mA3 allelic variants are placed at trapping sites.

Because the inclusion of exon 5 into mA3 message depends on the intron 4 TCCT repeat and the exon 5 G/C polymorphism at position 88 (G/C88), we screened additional inbred strains and wild mouse species for these sequence variations and for the presence or absence of exon 5 in the mA3 message. We sequenced exon 5 and segments of the flanking introns from 39 mice that represent different taxa or members of the same species trapped in different geographic locations (Table S1), as well as those from the inbred laboratory strains B10.A and A/WySn, prototypic strains with FV-restrictive and -permissive phenotypes [26], [27], [31], [32]. The complete dataset is shown in Figure S1. Genomic sequencing showed that A/WySnJ mice share the TCCT duplication and exon 5 C88 SNP with BALB/c, while B10.A mice are identical to B6 mice at both sites (Figure 9A); this is consistent with previous observations on mA3 expression levels and inclusion of exon 5 in these strains of mice [27]. Among the 39 wild mice, the TCCT duplication was found in 10 mice that possessed the exon 5 C88 SNP, and all of the 29 other mice with G88 had a single TCCT copy as representatively shown in Figure 9A. The observed linkage between the repeated TCCT and C88 is reasonable, as both are required for efficient expression of 5+ message from the BALB/c allele (Figures 4, 6, and 8). RNA samples from 23 of the above mice were typed for the splicing phenotype. The presence or absence of exon 5 in mA3 mRNA correlated with the above identified sequence polymorphisms: exon 5 was present only in 7 mice, all of which had the linked TCCT duplication and C88, consistent with our functional assays. On the other hand, there were at least 7 discrepancies with respect to exon 5 inclusion and the C14 SNP confirming that this SNP has no major role in exon 5 splicing.

The coding sequence for exon 5 was present in all 39 *Mus* DNA specimens, as well as in rat DNA specimens (GenBank EDM15775) as described previously [29]. There were frameshift mutations within exon 5 in *M. setulosus* and both *M. cervicolor* subspecies, all of which lack exon 5 in mA3 mRNA, and the exon 5-containing splice variant was found only in mA3 mRNA of *M. spretus* and 3 of the house mouse species (*M. domesticus*, *M. musculus*, *M. castaneus*) (Figure 9B). The wild mice were also tested for the presence or absence of another genetic variation associated with variable expression levels of mA3, the exon 2-associated xenotropic MuLV LTR insertion [37]. Results indicated that the presence of this LTR (LTR^+^), like the inclusion of exon 5 due to the above allelic variation (exon 5^+^), was introduced into the *Apobec3* locus at about the time of the house mouse radiation (Figure 9B). These two features are not found together in any mouse, and the LTR^+^ and exon 5^+^ variants are both found in mice classed as *M. musculus* or *M. domesticus*, while *M. castaneus* mice have both variants as well as the ancestral Δ5 LTR^−^ mA3 type.

To better understand this species distribution, we typed additional samples of European *M. domesticus* and *M. musculus* for the presence of the LTR and exon 5 (Figure 9C). The geographic distribution of the trapping sites for these 19 house mice and for the exon 5^+^ *M. spretus* indicated that the exon 5^+^ mA3 was characteristic of *M. domesticus* of north Africa and western Europe, whereas LTR^+^ mA3 was found in eastern Europe (*P* = 0.049 by Fisher's exact test). Four of the 9 eastern European *M. musculus* mice carried the *M. domesticus* variant, which is consistent with previous reports that gene flow across the hybrid zone is biased in the direction from *M. domesticus* to *M. musculus* ([42], for example). Additional samples identified as *M. domesticus* were trapped in the Americas (HAF, CL, WSA, PGN2, SC1, BQC, SAF, JJD, PERA; see Table S1), and these carried either the exon 5^+^ mA3 (like *M. domesticus*) or the LTR^+^ mA3 (like *M. musculus*); this is likely due to the fact that *Mus* are non-native species that were introduced into the Americas by passive transport, and although generally classed as *M. domesticus*, these mice show evidence of hybridization with other introduced house mouse species [43], [44].

## Discussion

Polymorphisms in mA3 are responsible for the fact that the B6 and C57BL/10 mouse strains are more restrictive to the replication of both beta- and gammaretroviruses than the BALB/c and A strains [25]–[29]. These differences in mA3 antiviral activities have been associated with sequence differences in the N-terminal region of mA3 [27], different levels of its mRNA expression [27], [29], [38], [39], and the presence or absence of exon 5 in mA3 mRNA and protein [27], although the relative importance of these three factors for antiviral activity has not been established. In the present study, we focused on the role of exon 5 and have shown that the Δ5 mRNA directs more efficient mA3 protein synthesis than the exon 5-containing message. We have also identified 2 genetic determinants responsible for the inclusion of this exon into mA3, a TCCT repeat in intron 4 and a C88 substitution in exon 5 relative to the B6 allele. We further showed that these two determinants were coordinately acquired within the last 0.5 million years by house mouse species of *Mus*.

Previous studies have recognized differences in mRNA expression levels of mA3 between B6 and BALB/c mice [27], [29], [38], [39]. Higher constitutive expression of mA3 mRNA is thought to contribute to better antiviral activity as suggested by the facts that higher levels of protein production result in more efficient incorporation of mA3 into viral particles [45] and that lipopolysaccharide (LPS)-induced enhancement of mA3 expression results in better restriction of MMTV *in vivo* [46]. It has also been shown that increased APOBEC3G and APOBEC3F expression is associated with lower viral load in rhesus macaques infected with simian immunodeficiency virus [47]. Higher protein levels of hA3G in CD14^+^ monocytes are also associated with HIV-1-exposed but uninfected status in humans [48]. The increased level of *Apobec3* gene transcription has been associated with the presence in the B6 allele of an intact MuLV LTR, a sequence capable of driving enhanced transcription [37]. While increased levels of the mA3 transcript could be solely responsible for the presently observed increase in mA3 protein levels in B6 mice (Figure 1D), it was also reported that, when driven by the same strong promoter, the B6 mA3 cDNA lacking exon 5 produced more protein product than the exon 5-containing BALB/c cDNA and resulted in more efficient incorporation into viral particles [27], [45]. Thus, it was unclear whether the experimentally observed increased translation was due to the lack of exon 5 in the B6 cDNA or to the other sequence differences that distinguish the two *Apobec3* alleles [27], [37], [38]. Here we have clearly shown that the Δ5 mRNA directs much more efficient protein synthesis than the 5+ mA3 mRNA regardless of other allelic differences (Figures 2 and 3), and the combination of the higher mRNA expression levels with preferential generation of the Δ5 isoform in mRNA splicing results in much higher mA3 protein levels in B6 in comparison with BALB/c mice (Figure 1).

The mechanisms by which the presence of exon 5 in the mRNA interferes with protein synthesis are currently unclear: however, predictions of the secondary structure for the mA3 mRNA suggest that the portion encoding exon 5 may form stable stem-loop structures (Figure S2), which might interfere with efficient translation. The predicted stem-loop structure is thermodynamically more stable for B6 than for BALB/c exon 5, and this portion is actually spliced out from the major transcript in B6 mice. It is possible, therefore, that the elimination of this exon from mA3 mRNA confers a functional advantage to B6 mice through more efficient translation of the Δ5 mRNA and resultant higher levels of mA3 protein expression. It should be noted, however, that introduction of this exon between the two CDDs does not physically disrupt the functional domains, as the 5+ product of the BALB/c allele still exerts deaminase activity and restricts AKV replication *in vitro* [29]. The possible effect of this 33-amino acid exon on interactions between the N-terminal and C-terminal CDDs and on mA3 oligomerization remains to be elucidated.

Our results also show that the ancestral *Mus Apobec3* gene locus lacked both the MuLV LTR and the functional polymorphisms that determine exon 5 inclusion (Figure 9). These two genetic features were acquired independently at about the time of the house mouse radiation 0.5–1.0 million years ago, and are found in distinct lineages of wild mice and different inbred strains of laboratory mice. It should be noted that all 4 house mouse species originated and diverged from an ancestral population on the Indian subcontinent that carries many of the alleles found in peripheral Eurasian populations [49], [50]. The observed distribution of the LTR^+^ and exon 5^+^ mA3 variants among the house mouse lineages is consistent with the appearance of these variants in this ancestral population. It is thus likely that *M. domesticus* progenitors carrying the exon 5^+^ *Apobec3* allele moved west to north Africa and western Europe, and *M. musculus* (LTR^+^) moved north to Russia, eastern Europe and north China. *M. castaneus*, which carries these two variants as well as the ancestral Δ5 LTR^−^ mA3, migrated east to Thailand and China. Only the ancestral mA3 type is found in *M. molossinus*, which is a natural hybrid of *M. musculus* and *M. castaneus* [44], and these Japanese mice presumably inherited their *Apobec3* gene from their *M. castaneus* progenitors.

Numerous studies indicate that host antiviral factors co-evolve with the pathogens they restrict, and this “arms race” is responsible for mutational changes in APOBEC3 in primates and mice as evidenced by detectable positive selection [37], [51]. The acquisition of the MuLV LTR that is associated with enhanced mA3 transcription in virus-infected mice also makes sense as an evolutionary adaptation to pathogen infection [37]. Thus, the B6 *Apobec3* allele has acquired two advantageous features that contribute to enhanced retrovirus restriction: high mRNA expression directed by the intron 2 LTR that is in linkage with the positively selected, more functional amino acid sequence in the N-terminal deaminase domain [27], [37]. The BALB/c allele, in contrast, acquired the polymorphisms that direct exon 5 inclusion as shown in the present study, along with coding sequence polymorphisms associated with lower antiviral activity [27]. The far-flung distribution of exon 5^+^ mA3 throughout western Europe (Figure 9C) is surprising, because this allele, having a reduced level of mA3 protein expression with inefficient restriction of MuLV replication at least *in vitro* [27], would seem to be evolutionarily deleterious. The observed distribution of exon 5^+^ mA3 suggests either that the exon 5^+^ variant provides sufficiently protective levels of antiviral activity in virus-infected mice and is therefore not subject to purifying selection, that these particular mice have not been exposed to significant challenge by mA3-sensitive pathogens, that this mA3 variant provides some other, unrecognized selective advantage, or that this allele is tightly linked to another advantageous polymorphism.

All 4 house mouse species possessing the exon 5^+^ genetic variation carry beta- and gammaretroviruses along with other retroelements that are subject to APOBEC3 restriction [25]–[30], [52]. In this regard, although the BALB/c mA3 is less antiviral than the product of the B6 allele, it does have measurable antiviral activity [29]. Further, analysis of 54 germline MuLV proviruses of three envelope types in the sequenced B6 genome demonstrated evidence of APOBEC3-mediated editing of the polytropic and modified polytropic, but not the xenotropic, proviruses [30]. The mA3-edited polytropic and modified polytropic viruses originated in *M. domesticus* [52], [53], mice that carry the exon 5^+^ mA3, suggesting that this mA3 variant may be effectively antiviral *in vivo*. Finally, in mA3-deficient B6 mice, the absence of this protein differentially affects FV replication depending on target cell types [54]. As cell type-dependent and pathogen-induced changes in mA3 expression may differ between mice of the ancestral Δ5 LTR^−^, exon 5^+^, and LTR^+^ variants, mice with the exon 5^+^ *Apobec3* allele may still restrict mA3-sensitive pathogens in critical target cells.

It is also possible that some mA3 polymorphisms have been selected for reasons other than their antiviral functions, and these functions may take precedence in mice not threatened with retrovirus assault. It is unlikely that differences in mA3 expression levels and/or its amino acid sequence provide a significant selective advantage in terms of normal development, survival, or fertility, as *Apobec3*-knockout mice show no increased propensity for tumor development or disease, and both male and female mA3-deficient mice were fertile [40]. On the other hand, the presence of active cytidine deaminases can have costs that may become more significant when endogenous and exogenous retroviral activity is low. Transgenic mice overexpressing mouse activation-induced deaminase (AID) or rabbit apolipoprotein B mRNA-editing enzyme catalytic polypeptide 1 (APOBEC1), two other members of the AID/APOBEC family of cytidine deaminases, developed neoplastic diseases and showed evidence of significant editing of various expressed genes [55], [56]. This suggests that when the retroviral threat is low, the consequences of possessing highly active mutagenic enzymes, like the Δ5 mA3 expressed in B6 mice, can outweigh their advantages as antiviral factors. In this regard, it should be noted that infectious MuLV has not been isolated from European *M. domesticus*, which carry the less highly expressed 5+ mA3, whereas the more active *Apobec3* alleles are found in all 3 of the other species of *Mus*, all of which have been found to carry infectious MuLVs [57].

Finally, it has also been observed that there is a similarly wide-spread distribution in humans of a deletion of a genomic segment harboring the *Apobec3b* gene locus [58]. At least one copy of this deletion is present in >40% of humans. The selective advantage of this genetic variant has not been determined, but its retention may suggest a possible advantage in reducing the genotoxic activity of this cytidine deaminase, or alternatively, it may be linked to positively selected variants in one or more of the 6 linked human APOBEC3 paralogues [59], [60]. Further comparative analyses of the geographic and species distributions of natural mouse pathogens and the various mA3 variants may help define previously unrecognized targets of APOBEC3-mediated restriction and provide further insight into the coevolution of pathogens and this host restriction factor.

## Materials and Methods

### Ethics statement

The studies utilizing laboratory animals were carried out in strict accordance with the Act on Welfare and Management of Animals of the government of Japan and the Regulations for the Care and Use of Laboratory Animals of Kinki University. The protocol was approved by the Institutional Animal Experimentation Committee of Kinki University School of Medicine (Permit Number: KAME-19-029). All surgery was performed under sodium pentobarbital anesthesia, and all efforts were made to minimize suffering. All studies involving wild mice were performed in compliance with the US Government Principles for the Utilization and Care of Vertebrate Animals used in Testing, Research, and Training; the Public Health Service Policy on Humane Care and Use of Laboratory Animals; The Animal Welfare Act and amendment laws; the Animal Care Policies of the USDA; The Guide for the Care and Use of Laboratory Animals (7th Edition; National Research Council); and the guidelines of the Committee on the Care and Use of Laboratory Animals under an NIAID-approved animal study protocol, and all studies and procedures were reviewed and approved by the Institutional Animal Care and Use Committee of the NIH (Permit Number: ASP LMM 1).

### Mice and mouse genomic DNA

C57BL/6NCrslc, BALB/cCrslc, and B10A/SgSnslc mice were purchased from Japan SLC, Inc., Hamamatsu, Japan. Breeding pairs of A/WySnJ mice were purchased from The Jackson Laboratory, Bar Harbor, ME. The mA3-deficient strain on the B6 background has been described [27], [40]. All laboratory mice were housed and bred in the Experimental Animal Facilities at Kinki University School of Medicine under specific pathogen-free conditions. The isolation of genomic DNA from spleens was carried out with DNeasy blood and tissue kit (Qiagen, Inc., Hilden, Germany) according to the manufacturer's instructions.

DNA and RNA were separately isolated from animals and cell lines developed from wild mice and wild mouse-derived breeding colonies or inbred strains (Table S1). Many wild-derived mice were obtained from M. Potter (National Cancer Institute, Bethesda, MD). CAST/Rp mice were obtained from R. Elliott (Roswell Park Cancer Institute, Buffalo, NY). Cells from some wild mouse species were obtained from J. Rodgers (Baylor College of Medicine, Houston, TX) and from J. Hartley, M. Lander or S. Chattopadhyay (National Institute of Allergy and Infectious Diseases, Bethesda, MD) [61], [62]. *M. cervicolor popaeus* mice and tissue samples were obtained from R. Callahan (National Cancer Institute). Mice or DNA samples of inbred lines of *M. castaneus* (CAST/EiJ) and *M. molossinus* were obtained from The Jackson Laboratory. *M. musculus* DNA samples were obtained from S. Chattopadhyay and H. Morse (National Institute of Allergy and Infectious Diseases). DNA samples from wild-trapped European *M. domesticus* were provided by M. Nachman (University of Arizona, Tucson). DNA samples from 5 wild-derived strains (BLG2, NJL, MSM, HMI, PGN2) were obtained from the National Institute of Genetics, Mishima, Japan. A set of *Nannomys* DNAs was obtained from Y. Cole and P. D'Eustachio (Departments of Biochemistry and Medicine, New York University, NY); these mice were classed into 4 species on the basis of skeletal features by J. T. Marshall (Smithsonian Natural History Museum, Washington, DC). DNA was isolated from cultured tail biopsies, spleen, or liver by standard protocols, and RNA was isolated from the spleen or liver using TRI-Reagent (Molecular Research Center, Cincinnati, OH) or by a guanidine chloride extraction method [63].

### Mouse *Apobec3* sequences and the prediction of mRNA secondary structures

DNA containing the mouse *Apobec3* exon 5 and associated intron sequences was amplified using either one of the following forward primers: 5′-GGACAATGGTGGCAGGCGATTC-3′, 5′-GCATCTTTGTGGATGGGG-3′, and the reverse primer 5-TCATTCCTCAATGCTCCTCC-3′. PCR products were cloned into pCR2.1-TOPO (Invitrogen, Carlsbad, CA) before sequencing. The *Apobec3* region was amplified from genomic DNA of B6, BALB/c, B10A, and A/WySnJ mice using the forward primer 5′-TTACAAATTTTAGATACCAGGATTCTAAGCTTCAGGAG-3′ and the reverse primer 5′-GTCCTTTATGTGGGTTCCAAGGACC-3′. PCR products were treated with ExoSAP-IT (USB, Cleveland, OH) and directly sequenced with the above reverse primer. To determine the BALB/c genomic sequence of the *Apobec3* intron 5, the BALB exon 5–6 and BALB intron 5-Δ3′ plasmids (Figure 5) were sequenced by using BigDye Terminator V3.1 Cycle Sequencing Kit (Applied Biosystems, Foster City, CA) and an ABI PRISM 3100 Genetic Analyzer (Applied Biosystems) using the following primers: T7 promoter forward, 5′- TAATACGACTCACTATAGGG-3′; and V5 reverse, 5′-CGTAGAATCGAGACCGAGGAGAGGGTTAGGGATAGGC-3′.

The secondary structure of a portion of the mA3 mRNA exon 5 was predicted with *mfold* [64], [65].

### Cell culture and DNA transfection

BALB/3T3 and human 293T cells were cultured in Dulbecco's modified Eagle medium supplemented with 10% heat-inactivated fetal bovine serum (Invitrogen). These cells were seeded at 1.0×10^5^/well in a well of 6-well plates one day prior to transfection. DNA transfection was performed by using Lipofectamine 2000 (Invitrogen) according to the manufacturer's protocols. Mouse spleen cells were harvested as described previously for protein and RNA extractions [27]. At least three independent transfection experiments were performed in this study and representative results are shown in the figures.

### Plasmid constructions

The expression plasmids, pFLAG-CMV2-*mA3^b^*Δ5 harboring the mA3 cDNA derived from the Δ5 transcript of the B6 allele, pFLAG-CMV2-*mA3^d^* harboring the cDNA derived from the 5+ transcript of the BALB/c allele, pFLAG-CMV2-*mA3^d^*Δ5 harboring the cDNA derived from the Δ5 transcript of the BALB/c allele, and control pFLAG-CMV2-GFP have been described [27]. The genomic DNA encoding the mA3 exon 5 was amplified from B6 genome with the following primers: 5′-ACCTTGCTACATCTCGGTCCCTTCCAGC-3′ and 5′-CTGCCCTCCACCCAGAACCTCGTCTCTGG-3′. The above pFLAG-CMV2-*mA3^b^*Δ5 was used as a PCR template with primers 5′-GCGAATGGACCCGCTAAGTGAAGAGG-3′ and 5′-CTCAGAATCTCCTGAAGCTTAGAATCCTGG-3′ to amplify the linearized plasmid with a gap between exons 4 and 6. The two PCR products above were treated with T4 polynucleotide kinase (TAKARA Bio, Otsu, Japan) and fused by using the DNA Ligation Kit ver.2.1 (TAKARA Bio) to construct the plasmid pFALG-CMV2-*mA3^b^*, which expresses the 5+ mA3 cDNA derived from the B6 allele.

B6 or BALB/c genomic fragments harboring exons 4–7 and the intervening introns were amplified by PCR using either the B6 or BALB/c genomic DNA as a template and the common primers 5′-CACCAATTTAAAAAGTGTTGGAAGAAG-3′ and 5′-GTGGGAGGTCCATGACGTCCACCAGGATCCC-3′. Each amplified DNA product was cloned into a pcDNA3.2/V5/GW/D-TOPO cloning vector (Invitrogen) and designated as B6 or BALB exon 4–7, respectively. The above B6 or BALB exon 4–7 was used as a template with the primers 5′-CACCACCTTGCTACATCTCGGTCC-3′ and 5′-GCAGAGATGCTTGACTCGTTGGTTG-3′ or 5′-CACCACCTTGCTACATCCCGGTCC-3′ and 5′-GCAGAGATGCTTGACTCGTTGGTTG-3′, respectively, for the amplification of B6 or BALB/c exons 5 and 6 and the intervening intron 5. Each PCR product was cloned into the pcDNA3.2/V5/GW/D-TOPO vector and designated as B6 or BALB exon 5–6. The DNA fragments harboring sequentially deleted *Apobec3* intron 5 were prepared by PCR using either one of the above B6 or BALB exon 5–6 plasmids as a common template with the primer pairs A–F listed in Table S2. Each amplified DNA product was cloned into the pcDNA3.2/V5/GW/D TOPO vector to generate the expression plasmid shown in Figure 5B.

Reciprocal chimeras between the above B5 and BALB exon 5–6 plasmids were generated by amplifying a linearized plasmid DNA lacking the 3′ intron 5 and exon 6 using primer pair G, and by amplifying the insert fragment using primer pair H.

Site-directed mutagenesis was performed by employing the QuikChange Site-Directed Mutagenesis Kit (Stratagene, La Jolla, CA) using the following templates and primers, listed separately in Table S2: BALB 100bp intron 5 (3′-100bp) plasmid was used as a common template for the preparation of BALB C14T, BALB C88G, and BALB C153GG163A with primer pairs I, J, K, respectively: B6 100bp intron 5 (3′-100bp) plasmid was used as a template to make B6 T14C or B6 G88C with primer pair L or M, respectively. The resultant B6 T14C plasmid was used as a template with the same primer pair M employed for the generation of B6 G88C to make B6 T14C G88C. B6 T14C G88C was then used as a template for the generation of B6 T14C G88C G153C A163G by using primer pair O. Similarly, the above-used primer pairs were also utilized for the generation of B6 exon 5–6 T14C, B6 exon 5–6 T14C G88C, and B6 exon 5–6 T14C G88C G153C A163G mutants by using B6 exon 5–6 as a template. The B6 exon 5–6 plasmid was also used as a template with primer pairs M, P, and Q for making B6 exon 5–6 G88C, B6 exon 5–6 G153C, and B6 exon 5–6 A163G, respectively.

To make BALB ΔTCCT, 4 consecutive nucleotides within the repeat sequence, TCCT, were deleted from the BALB exon 4–7 plasmid by mutagenesis using primer pair R, listed in Table S2. BALB ΔTCCT or BALB exon 4–7 plasmid was used as a template with primer pair S to make BALB C741T ΔTCCT or BALB C741T, respectively. To introduce the TCCT sequence by mutagenesis and make B6+TCCT, B6 exon 4–7 plasmid was used as a template with primer pair T. B6+TCCT or B6 exon 4–7 was used as a template with primer pair U to generate B6 T741C+TCCT or B6 T741C, respectively. Similarly, the above produced B6 exon 4–7+TCCT or BALB exon 4–7 ΔTCCT plasmid was used as a template and G88C or C88G substitution was introduced with primer set M or J, respectively.

All resultant plasmids were entirely sequenced by using BigDye Terminator V3.1 Cycle Sequencing Kit with an ABI PRISM 3100 Genetic Analyzer. In order to normalize the transfection efficiency, a plasmid expressing the luciferase gene, p*luc,* based on the expression vector pGL3 (Promega, Madison, WI), was utilized. All the primers used in this study were purchased from Operon Biotechnologies, Tokyo, Japan.

### Quantitative real-time PCR assays for endogenous mA3 transcripts

For the quantification of mA3 transcripts in mouse spleens, total RNA was extracted from each spleen with RNeasy Mini Kit (Qiagen). The RNA was then subjected to reverse transcription with PrimeScript RT reagent Kit (TAKARA Bio). Real-time PCR reactions were carried out with SYBR Premix Ex Taq II (TAKARA Bio) on an Applied Biosystems 7900HT Fast Real-Time PCR System (Applied Biosystems) with two different sets of *Apobec3*-specific primers: set 1, 5′-GTGTTGGAAGAAGTTTGTGG-3′ (primer a) and 5′-CCTGAAGCTTAGAATCCTGG-3′ (primer b); and set 2, 5′-TTACAAATTTTAGATACCAGGATTCTAAGCTTCAGGAG-3′ (primer c) and 5′-TTGGTTGTAAAACTGCGAGTAAAATTCCTCTTCAC-3′ (primer d). The data were normalized with expression levels of β-actin mRNA to obtain ΔCt values, and ΔΔCt values were calculated.

### Detection and quantification of the *mA3* transcripts and their splicing products

For the detection of endogenous mA3 transcripts in mouse spleen cells, the RT products and primer sets used for the real-time PCR were also utilized for PCR using KOD Dash DNA polymerase (Toyobo, Osaka, Japan). The PCR products were separated by 1% agarose gel electrophoresis and detected by staining with ethidium bromide. For splicing assays, BALB/3T3 cells were transfected with 1 µg of each plasmid harboring a genomic DNA fragment and 0.5 µg of p*luc* to normalize the transfection efficiency. Total RNA was extracted from the transfected cells using the RNeasy Mini Kit at 24 hours post-transfection. The total RNA was treated with DNase I, reverse transcribed with SuperScript III First Strand Synthesis System (Invitrogen), and the resultant cDNA was subjected to PCR detection using KOD Dash DNA polymerase with the following primers: 5′-TAATACGACTCACTATAGGG-3′ (primer g) and 5′-CGTAGAATCGAGACCGAGGAGAGGGTTAGGGATAGGC-3′ (primer h), designed to hybridize the T7 promoter and V5 tag regions of the vector, respectively. Primer i, 5′-GGTCTCCCAGAGACGAGGTTCTG-3′, was used to detect only the exon 5-containing transcript. For real-time PCR quantification of the *mA3* transcripts containing exon 5, primer j (5′-GTGGATGAAGAGCTGGAAGGGACCG-3′) was used along with the above primer g. Transcripts containing exon 6 were similarly quantified by using primer k (5′-CAACCAACGAGTCAAGCATCTCTGC-3′) and the above primer h.

For RT-PCR detection of mA3 mRNA in cells transfected with an mA3 expression plasmid, the same RT product as was used for real-time PCR analyses was utilized with the above-described primer set 1 (a–b). The RT products made by PrimeScript RT reagent Kit were supposed to be relatively short in length because a mixture of oligo-dT and random 6-mers were used as primers in the RT reaction. Thus, in order to detect the full-length and Δ5 transcripts, the following primers, 5′-GGGAATTCGATGGGACCATTCTGTCTGGGATGCAGCCATCGC-3′ (primer e) and 5′-GGGTCGACTCAAGACATCGGGGGTCCAAGCTGTAGGTTTCC-3′ (primer f), were used along with newly synthesized RT products generated by the SuperScript III First Strand Synthesis System (Invitrogen). To quantify the mA3 transcripts in 293T cells transfected with an mA3 expression plasmid, one-third of the transfected cells were used for total RNA isolation using the RNeasy Mini Kit at 24 hours after transfection. The purified RNA was treated with 5 units of DNase I (TAKARA Bio) for 1 hour at 37°C to digest the transfected DNA and then reverse transcribed with the PrimeScript RT reagent Kit. The real-time PCR was performed as described above.

For RT-PCR analyses of mA3 transcripts generated by the *in vitro* transcription/translation system, the reaction was stopped by the addition of lysis buffer included in RNeasy Mini Kit after 30 or 60 min of incubation, and the transcribed products were purified with the above kit. After the treatment of the purified RNA with DNase I, the RT-PCR reaction was carried out with the above primer set 1. After electrophoresis, gel images were recorded with a FluorChem™ IS-8900 transilluminator and band intensities were analyzed with AlphaEase FC Stand Alone software (Alpha Innotech, San Leandro, CA).

*Apobec3* splicing patterns for wild-derived mice were identified by RT-PCR to amplify a segment of mA3 RNA spanning exon 5 from total RNA using forward primer 5′-GGACCATTCTGTCTGGGATGCAGCCATCG-3′ and reverse primer 5′-GGTTGTAAAACTGCGAGTAAAATTCC-3′.

### Luciferase assays

Luciferase assays for normalization of transfection efficiencies were performed by utilizing the Luciferase Assay System (Promega). The enzymatic activities were measured by Wallac 1420 ARVO™ MX-2 Multilabel Counter (Perkin Elmer).

### Pre-absorption of the antibody

A spleen from an *Apobec3* knock-out mouse [40] was homogenized in 1ml ice-cold phosphate-buffered saline (PBS). Four ml of ice-cold acetone was added to the homogenate, mixed, and incubated on ice for 30 min. The lysate was centrifuged at 10,000×*g* at 4°C for 10 min. The pellet was washed with ice-cold acetone once and dried completely at room temperature to make spleen extract powder. Two µl of anti-APOBEC3 NT antibody specific for the N-terminal portion of mA3 (Millipore, Billerica, MA) was added to 0.5ml of KTBT buffer (50 mM Tris-HCl, pH 7.5, 150 mM NaCl, 10 mM KCl, 1% Triton X-100) containing 10% (w/v) unimmunized sheep serum as a carrier, into which 3mg of the spleen extract powder was dissolved. The mixture was incubated overnight at 4°C with gentle rotation. After centrifugation at 10,000×*g* at 4°C for 10 min, the supernatant was used as a pre-absorbed anti-mA3 antibody.

### Immunoblotting

Western blotting analyses were conducted as described previously [27] with some modifications. Briefly, proteins were extracted with a lysis buffer (1% Nonidet P-40, 25 mM Tris-HCl, pH 7.5, 140 mM NaCl, 1 mM EDTA, 10 mM Na_4_P_2_O_7_) containing protease inhibitors from Complete, Mini, EDTA-free Protease Inhibitor Cocktail Tablets (Roche Applied Science, Mannheim, Germany) and a phosphatase inhibitor, PhosSTOP (Roche Applied Science). Total protein concentrations were determined by Bradford assay (Nacalai Tesque, Kyoto, Japan) and the extracts were mixed with sodium dodecyl sulfate (SDS)-polyacrylamide gel electrophoresis (PAGE) sample buffer and heated at 95°C for 5 min. The proteins were separated by SDS-PAGE, transferred to Immobilon-P membrane (Millipore), and the blotted membranes were blocked with 5% (w/v) skim milk (Wako Pure Chemicals, Osaka, Japan) in Tris-buffered saline with 0.05% Tween 20 (TBST). The blocked membranes were incubated with the primary antibody at 4°C overnight. Membranes were then washed with TBST and incubated with horse radish peroxidase (HRP)-conjugated secondary antibody for 2 hours at room temperature, washed again with TBST, and the bound antibodies were detected using ECL plus reagent (GE Healthcare, Tokyo, Japan). The images were captured with a LAS-1000 Plus (Fujifilm, Tokyo, Japan) and the band intensities evaluated with Image Gauge ver. 3.12 (Fujifilm). A 1/5 dilution of the above-mentioned pre-absorbed mA3 antibody was made with IMMUNO SHOT (COSMO BIO, Tokyo, Japan) and was used as a primary antibody. Anti-FLAG M2 monoclonal antibody (mAb) (Sigma-Aldrich), anti-IκBα mAb (L35A5) (Cell Signaling Technology, Beverly, MA), anti-actin antibody (C-11) (Santa Cruz, CA), and the His-probe (H-15) (Santa Cruz) were diluted at 1∶1000 with TBST. HRP-conjugated rabbit anti-mouse IgG (Zymed, South San Francisco, CA) and HRP-conjugated Goat anti-rabbit IgG antibodies (Invitrogen) were also diluted at 1∶1000 and used as secondary antibodies to detect each appropriate primary antibody.

### Assessment of mA3 protein stability

1.0×10^5^ of 293T cells were transfected with 0.1 µg of an mA3 expression plasmid, 0.01 µg pFLAG-CMV2-GFP, and 0.1 µg of p*luc*. After 24h, the cells were treated with 10 µg/ml of cycloheximide or its solvent dimethyl sulfoxide (DMSO) as a control for 0, 2, or 4 hours. The cells were washed and resuspended in PBS. One-tenth of the cell suspension was subjected to luciferase assays to normalize transfection efficiencies, and the remaining cells were dissolved in the SDS-PAGE sample buffer. The normalized amount of cell lysates were separated by SDS-PAGE followed by immunoblotting as described above.

### *In vitro* transcription and translation

The FLAG-mA3 cDNA was amplified with highly proofreading Pfu Turbo DNA Polymerase (Stratagene) from the plasmids pFLAG-CMV-*mA3^d^* and pFLAG-CMV-*mA3^d^*Δ*5* [27], with a forward primer harboring the T7 promoter sequence, 5′-GGATCCTAATACGACTCACTATAGGGAACAGCTGGGATGGGACCATTCTGTCTGGGATGC-3′ and a reverse primer harboring the His-Tag sequence, 5′-TCAATGGTGATGGTGATGATGAGCAGCAGCAGACATCGGGGGTCCAAGCTGTAGG-3′. The PCR products were subjected to reactions for *in vitro* transcription and translation using TNT T7 Quick for PCR DNA (Promega) according to the manufacturer's protocol. Half of the generated products were mixed with the SDS-PAGE sample buffer and analyzed by immunoblotting. The remaining products were used for RNA purification with RNeasy Mini Kit (Qiagen) followed by RT-PCR to detect mA3 transcripts.

## Supporting Information

Figure S1 **Nucleotide sequence of the genomic region encoding mA3 exon 5 and segments of flanking introns from several laboratory mouse strains and wild mouse species.** Mouse *Apobec3* exon 5 and the flanking introns from 39 mice that represent different taxa or members of the same species trapped in different geographic locations, as well as those from the inbred laboratory strains BALB/c, B10.A, and A/WySn, were sequenced and aligned with the corresponding B6 sequence. The exon 5 and six key polymorphic regions, C/T741 and TCCT repeat in intron4, C/T14 and C/G88 in exon5, and C/G153 and A/G163 in intron 5, are indicated. Accession numbers for all newly obtained sequence data are also provided in this figure.
(PDF)

Figure S2 **Possible stem-loop structures predicted from the mA3 intron 5 mRNA sequence.** The mRNA secondary structures of exon 5 encoded by the B6 and BALB/c alleles were predicted by using the mfold [64], [65]. Polymorphic nucleotides within this exon, U/C at position 14 and G/C at position 88, are indicated.
(PDF)

Table S1 **Designations and sources of wild-derived mice, their cells, and DNA samples.**
(PDF)

Table S2 **Primers used to generate intron 5 deletion mutants and chimeras, for exon 5/intron 5 nucleotide substitutions, and for modification of TCCT repeat and T/C 741 SNP in intron 4.**
(PDF)
