# Supplementary material for: Two Genetic Determinants Acquired Late in Mus Evolution Regulate the Inclusion of Exon 5, which Alters Mouse APOBEC3 Translation Efficiency
Source: PLoS Pathog. 2012 Jan 19;8(1):e1002478. doi: 10.1371/journal.ppat.1002478 (PMC3262013; doi:10.1371/journal.ppat.1002478)
Supplement: Figure S1 — Nucleotide sequence of the genomic region encoding mA3 exon 5 and segments of flanking introns from several laboratory mouse strains and wild mouse species. Mouse Apobec3 exon 5 and the flanking introns from 39 mice that represent different taxa or members of the same species trapped in different geographic locations, as well as those from the inbred laboratory strains BALB/c, B10.A, and A/WySn, were sequenced and aligned with the corresponding B6 sequence. The exon 5 and six key polymorphic regions, C/T741 and TCCT repeat in intron4, C/T14 and C/G88 in exon5, and C/G153 and A/G163 in intron 5, are indicated. Accession numbers for all newly obtained sequence data are also provided in this figure. (PDF) [file ppat.1002478.s001.pdf]

Figure S1. Nucleotide sequence of the genomic region encoding mA3 exon 5 and segments of flanking introns from several laboratory mouse strains and wild mouse species listed in Table S1. The exon 5 sequence is in red and six key polymorphic regions are highlighted with yellow: C/T741 and TCCT repeat in intron4, C/T14 and C/G88 in exon5, C/G153 and A/G163 in intron 5. The C57BL/6 [AC113595] and rat [CH473950] sequences are added for comparison. All newly obtained sequence data have been submitted to GenBank (accession number is shown at the end of each sequence). BALB/c sequence data including those for intron 5 have been submitted to DDBJ [accession numbers: AB646261-AB646265].

```
BALB/c                     GCATCTTTGTGGATGGGGAGCCCAGAGTCCTCACTCTGGTT-ACCAACACCCCAGGAGAATCCCT
A/WySnJ                    .................................................................
M.musculusSKIVE            -................................................................
M.musculusVEJ              .................................................................
M.castaneusHMI             .................................................................
M.castaneusCASTEi          .................................................................
M.domesticusSC1            .................................................................
M.domesticusSAF            .................................................................
M.domesticusJJD            .................................................................
M.domesticusBQC            .................................................................
M.domesticusPosch2         .................................................................
M.domesticusABUR           .................................................................
M.spretus                  .................................................................
C57BL/6                    ...........................................T.....................
B10.A                      ...........................................T.....................
M.musculusNJL              ...........................................T.....................
M.musculusCzI              ...........................................T.....................
M.musculusCzII             ...........................................T.....................
M.musculusBLG2             ...........................................T.....................
M.castaneusCasLi           ...........................................T.....................
M.castaneusCASTN           ...........................................T.....................
M.castaneusCASTRp          ...........................................T.....................
M.domesticusPGN2           ...........................................T.....................
M.domesticusCL             ...........................................T.....................
M.domesticusHAF            ...........................................T.....................
M.domesticusWSA            ...........................................T.....................
M.domesticusGREECE         ...........................................T.....................
M.molossinusMSM            ...........................................T.....................
M.molossinusMOL            ...........................................T.....................
M.molossinusMOLG           ...........................................T.....................
M.spicilegus               .................................................................
M.terricolor(dunni)        .................................................................
M.cervicolorpopaeus        .................................................................
M.cervicolorcervicolor     .................................................................
M.cookii                   .................................................................
M.caroli                   .................................................................
M.gratus                   .....................................................T...........
M.minutoides               .................C.......................T...........T...........
M.setulosus                .........................................T...........T...........
M.triton                   .............................................G.......T...........
M.saxicola                 .............................T...................................
M.shortridgei              ................................G.........G....................T.
M.pahari                   ................................G.........G....................T.
R.norvegicus               .TC.................TA.........TG.C.......G...G.............C..T.
```

```
BALB/c                     GACCATATCCTTCCTGGCCCCC-TCTTCCATTT-CCAGACCTTGCTACATCCCGGTCCCTTCCAGC
A/WySnJ                    ..................................................................
M.musculusSKIVE            ..................................................................
M.musculusVEJ              ..................................................................
M.castaneusHMI             ..................................................................
M.castaneusCASTEi          ..................................................................
M.domesticusSC1            ..................................................................
M.domesticusSAF            ..................................................................
M.domesticusJJD            ..................................................................
M.domesticusBQC            ..................................................................
M.domesticusPosch2         ..................................................................
M.domesticusABUR           ..................................................................
M.spretus                  ..................................................................
C57BL/6                    ...........----....................................T..............
B10.A                      ...........----....................................T..............
M.musculusNJL              ...........----....................................T..............
M.musculusCzI              ...........----....................................T..............
M.musculusCzII             ...........----....................................T..............
M.musculusBLG2             ...........----....................................T..............
M.castaneusCasLi           ...........----...................................................
M.castaneusCASTN           ...........----...................................................
M.castaneusCASTRp          ...........----...................................................
M.domesticusPGN2           ...........----....................................T..............
M.domesticusCL             ...........----....................................T..............
M.domesticusHAF            ...........----....................................T..............
M.domesticusWSA            ...........----....................................T..............
M.domesticusGREECE         ...........----....................................T..............
M.molossinusMSM            ...........----....................................T..............
M.molossinusMOL            ...........----....................................T..............
M.molossinusMOLG           ...........----....................................T..............
M.spicilegus               ...........----...................................................
M.terricolor(dunni)        ...........----.......C..............................A............
M.cervicolorpopaeus        ...A.......----...T...............................................
M.cervicolorcervicolor     ...A.......----...T...............................................
M.cookii                   ...........----...T...............................................
M.caroli                   ...........----..................................GT.G........T....
M.gratus                   ...........----..................T................T.T.............
M.minutoides               ...........----..................T................T.T.............
M.setulosus                ....G......----..................T...........G....T.T.A...........
M.triton                   ...........----..................T................T.T.........T...
M.saxicola                 ...........----.....G............T................................
M.shortridgei              ...........----..T..T............T............T.....T..G..........
M.pahari                   ...........----..T..T............T............T.....T..G..........
R.norvegicus               .......C...----...AT.............T.T.......A........T..GT........T
```

BALB/c	TCTTCATCCA-CTCTGTCAAATATCTGTCTAACAAAAGGTCTCCCAGAGACGAGGTTCTGCGTGG
A/WySnJ	....
*M.musculus*SKIVE	....
*M.musculus*VEJ	....
*M.castaneus*HMI	....
*M.castaneus*CASTEi	....
*M.domesticus*SC1	....
*M.domesticus*SAF	....
*M.domesticus*JJD	....
*M.domesticus*BQC	....
*M.domesticus*Posch2	....
*M.domesticus*ABUR	....
M.spretus	....
C57BL/6	....G....
B10.A	....G....
*M.musculus*NJL	....G....
*M.musculus*CzI	....G....
*M.musculus*CzII	....G....
*M.musculus*BLG2	....G....
*M.castaneus*CasLi	....T......G....
*M.castaneus*CASTN	....T......G....
*M.castaneus*CASTRp	....K......G....
*M.domesticus*PGN2	....G....
*M.domesticus*CL	....G....
*M.domesticus*HAF	....G....
*M.domesticus*WSA	....G....
*M.domesticus*GREECE	....G....
*M.molossinus*MSM	....G....
*M.molossinus*MOL	....G....
*M.molossinus*MOLG	....G....
M.spicilegus	....G....
M.terricolor(dunni)	.....G...........TT................................A........G....
M.cervicolorpopaeus	....................................T.-.....................G....
M.cervicolorcervicolor	....................................T.-.....................G....
M.cookii	....G....
M.caroli	....G....
M.gratus	......C...G....
M.minutoides	......C.....................................T...............G....
M.setulosus	......C...A...G....
M.triton	......C......G.C.....................A......................G....
M.saxicola	......C...G....
M.shortridgei	......C............G..G.................G......G..T.........G....
M.pahari	......C............G..G.................G......G..T.........G....
R.norvegicus	.A....C.....C.........G.........T....C............T.......A.G-...

```
BALB/c                      AGGGCAGGTGAGCCGTGGGGACTGGGGACTGAATGAAACAGAGTGGCAGGGTACACTTACCTAAG
A/WySnJ                     .................................................................
M.musculusSKIVE             .................................................................
M.musculusVEJ               .................................................................
M.castaneusHMI              .................................................................
M.castaneusCASTEi           .................................................................
M.domesticusSC1             .................................................................
M.domesticusSAF             .................................................................
M.domesticusJJD             .................................................................
M.domesticusBQC             .................................................................
M.domesticusPosch2          .................................................................
M.domesticusABUR            .................................................................
M.spretus                   .................................................................
C57BL/6                     ............................................................G....
B10.A                       ............................................................G....
M.musculusNJL               ............................................................G....
M.musculusCzI               ............................................................G....
M.musculusCzII              ............................................................G....
M.musculusBLG2              ............................................................G....
M.castaneusCasLi            ............................................................G....
M.castaneusCASTN            ............................................................G....
M.castaneusCASTRp           ............................................................G....
M.domesticusPGN2            ............................................................G....
M.domesticusCL              ............................................................G....
M.domesticusHAF             ............................................................G....
M.domesticusWSA             ............................................................G....
M.domesticusGREECE          ............................................................G....
M.molossinusMSM             ............................................................G....
M.molossinusMOL             ............................................................G....
M.molossinusMOLG            ............................................................G....
M.spicilegus                .............................................T...................
M.terricolor(dunni)         ............................................................G....
M.cervicolorpopaeus         ...........................................CA..............TG....
M.cervicolorcervicolor      ...........................................CA..............TG....
M.cookii                    ...........................................C...............TG....
M.caroli                    ...........................................................TG....
M.gratus                    ..T......A.A.......A..........................T............TG....
M.minutoides                ..T......A.........A......................................-------
M.setulosus                 ..T......A.C.......A.......................................TG....
M.triton                    ..TA.....AC.....................G..........................TG....
M.saxicola                  ..T........................................................TG....
M.shortridgei               .AT..............................C...G...G..A.A....C..T..C.TG....
M.pahari                    ..T..............................C...G...G..A.A....C..T..C.TG....
R.norvegicus                G.T..........T...A...........A...CAG..TG.....A.............TG....
```

		GenBank accession No.
BALB/C	AGGCCGGGGG------AGGAGCATTGAGGAATGA	[DDBJ AB646261-AB646265]
A/WySnJ	....	JN227718
*M.musculus*SKIVE	....	JN227713
*M.musculus*VEJ	....	JN227694
*M.castaneus*HMI	....	JN227687
*M.castaneus*CASTEi	....	JN248723
*M.domesticus*SC1	....	JN227710
*M.domesticus*SAF	....	JN227708
*M.domesticus*JJD	....	JN227707
*M.domesticus*BQC	....	JN227706
*M.domesticus*Posch2	....	JN227697
*M.domesticus*ABUR	....	JN227695
M.spretus	....	JN227696
C57BL/6	.....A............................	AC113595
B10.A	.....A............................	JN227717
*M.musculus*NJL	.....A............................	JN227686
*M.musculus*CzI	.....A..................----------	JN227699
*M.musculus*CzII	.....A............................	JN248725
*M.musculus*BLG2	.....A............................	JN227684
*M.castaneus*CasLi	....	JN227716
*M.castaneus*CASTN	....	JN248722
*M.castaneus*CASTRp	.....R............................	JN248724
*M.domesticus*PGN2	.....A............................	JN227683
*M.domesticus*CL	.....A............................	JN227709
*M.domesticus*HAF	.....A............................	JN227711
*M.domesticus*WSA	.....A............................	JN227712
*M.domesticus*GREECE	.....A............................	JN248726
*M.molossinus*MSM	.....A............................	JN227685
*M.molossinus*MOL	.....A............................	JN227701
*M.molossinus*MOLG	.....A................------------	JN227700
M.spicilegus	....	JN227688
M.terricolor(dunni)	....	JN227689
M.cervicolorpopaeus	....T.............................	JN227693
M.cervicolorcervicolor	....T.............................	JN227698
M.cookii	....	JN227691
M.caroli	....T.............................	JN227690
M.gratus	......C...GGTGGG..................	JN227702
M.minutoides	----------------------------------	JN227715
M.setulosus	....T.....G....G..................	JN227703
M.triton	.....ACC..AG..GG..................	JN227692
M.saxicola	.....A............................	JN227704
M.shortridgei	....T.............................	JN227705
M.pahari	....	JN227714
R.norvegicus	.....................GT........C..	CH473950
