# Supplementary material for: Two Genetic Determinants Acquired Late in Mus Evolution Regulate the Inclusion of Exon 5, which Alters Mouse APOBEC3 Translation Efficiency
Source: PLoS Pathog. 2012 Jan 19;8(1):e1002478. doi: 10.1371/journal.ppat.1002478 (PMC3262013; doi:10.1371/journal.ppat.1002478)

Figure S2. Possible stem-loop structures predicted from the mA3 intron 5 mRNA sequence. The mRNA secondary structures of exon 5 encoded by the B6 and BALB/c alleles were predicted by using the *mfold* [64, 65]. Polymorphic nucleotides within this exon, U/C at position 14 and G/C at position 88, are in red.

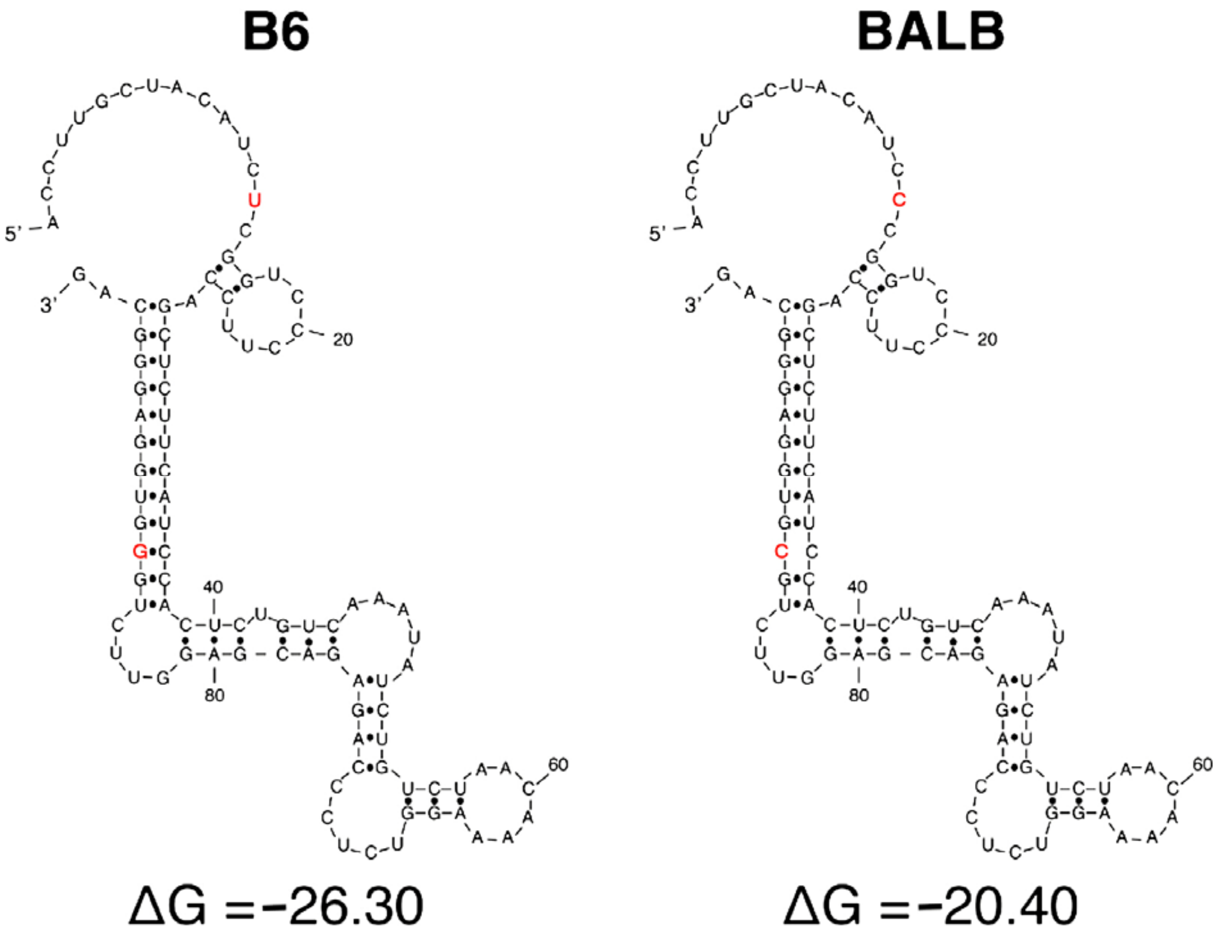

Supplement: Figure S2 — Possible stem-loop structures predicted from the mA3 intron 5 mRNA sequence. The mRNA secondary structures of exon 5 encoded by the B6 and BALB/c alleles were predicted by using the mfold [64], [65]. Polymorphic nucleotides within this exon, U/C at position 14 and G/C at position 88, are indicated. (PDF) [file ppat.1002478.s002.pdf]
