# Supplementary material for: Two Genetic Determinants Acquired Late in Mus Evolution Regulate the Inclusion of Exon 5, which Alters Mouse APOBEC3 Translation Efficiency
Source: PLoS Pathog. 2012 Jan 19;8(1):e1002478. doi: 10.1371/journal.ppat.1002478 (PMC3262013; doi:10.1371/journal.ppat.1002478)
Supplement: Table S1 — Designations and sources of wild-derived mice, their cells, and DNA samples. (PDF) [file ppat.1002478.s003.pdf]

Table S1. Designations and sources of wild-derived mice, cells and DNAs.

Subgenus	Species /subspecies	Previous species name or other designation	Geographic location	Type	Source (see Materials and Methods)
Coelomys	*pahari***			cells	Rodgers
Pyromys	*shortridgei***			cells	Rodgers
	*saxicola**		Mysore, India	mice	Potter
Nannomys	*minutoides***			cells	Rodgers
	*setulosus***		Nairobi, Kenya	cells	Rodgers
	*gratus**		Fort Portal, Uganda	DNA	D'Eustachio
	*triton**		Kanyawara, Uganda	DNA	D'Eustachio
Mus	*cervicolor cervicolor**		Loei Province, Thailand	mice	Potter
	*cervicolor popaeus***		Chonburi Province, Thailand	mice, tissue	Callahan
	*cookii**		Tak Province, Thailand	mice	Potter
	*caroli***		Chonburi Province, Thailand	mice	Potter
	*terricolor***	*dunni*	India	cells	Lander
	*spicilegus***	*hortulanus*		cells	Rodgers
	*spretus***		Puerto Real, Spain	mice	Potter
	*castaneus**	CAST/N	Thailand	mice	Potter
	*castaneus**	CAST/EiJ	Thailand	mice	Jackson Laboratory
	*castaneus**	CAST/Rp		mice	Roswell Park Cancer Inst.
	*castaneus***	CAS/Li		mice	Potter
	*castaneus**	HMI	Hemei, Taiwan	DNA	Natl. Inst. Genetics, Japan
	molossinus	MOLF/EiJ, MOLG/DnJ**	various in Japan	DNA	Jackson Laboratory
	*molossinus**	MSM	Mishima, Shizuoka, Japan	DNA	Natl. Inst. Genetics, Japan; Jackson Laboratory

	*molossinus***	MOL/Li	Kyushu, Japan	mice	Potter
	*musculus***	Skive	Skive, Denmark	mice	Potter
	*musculus***	CzI	Morovia, Czechoslovakia	mice	Potter
	*musculus***	CzII	Slovakia, Czechoslovakia	mice	Potter
	*musculus**	VEJ	Vejrumbro, Denmark	mice	Potter
	*musculus**	NJL	Northern Jutland,Denmark	DNA	Natl. Inst. Genetics, Japan
	*musculus**	BLG2	Toshevo, Bulgaria	DNA	Natl. Inst. Genetics, Japan
	musculus		Belgrade, Yugoslavia	DNA	Chattopadhyay, Morse
	musculus		Brno, Czechoslovakia	DNA	Chattopadhyay, Morse
	musculus		Viborg, Denmark	DNA	Chattopadhyay, Morse
	*domesticus***	CL (Centreville Lite)	Centreville, Maryland	mice	Potter
	*domesticus***	WSA (Watkins Star)	Watkins Farm	mice	Potter
	*domesticus***	HAF (Havens Farm)	Davidsonville, Maryland	mice	Potter
	*domesticus***	JJD (J.J. Downs)	Ridgely, Maryland	mice	Potter
	*domesticus***	SAF (Sanner's Farm)	Davidsonville, Maryland	mice	Potter
	*domesticus***	SC-1	California	cells	Hartley
	*domesticus**	BQC	Bouquet Canyon, California	mice	Potter
	*domesticus**	PGN2	Pegion, Canada	DNA	Natl. Inst. Genetics, Japan
	domesticus	PERA/EiJ	Rimac Valley, Peru	DNA	Jackson Laboratory
	*domesticus***	*poschiavinus* (Posch-2)	Zalende, Switzerland	mice	Potter
	*domesticus**	ABUR	Abu Rawash, Egypt	mice	Potter
	domesticus		Italy, Region Lazio	DNA	Nachman
	*domesticus**		Peleponissos, Greece	DNA	Nachman

	domesticus		Mallorca Island, Spain	DNA	Nachman
	domesticus		Catalunya, Spain	DNA	Nachman
	domesticus		Birmingham, England	DNA	Nachman
	domesticus		Papa Westray, Orkney	DNA	Nachman

*Sequenced for exon5 and flanking introns; ** sequenced and typed for splicing pattern. The additional mice were typed by PCR for the presence or absence of MuLV LTR within intron 2. MOLF and PERA mA3 sequences were obtained from [38].
