# Supplementary material for: Two Genetic Determinants Acquired Late in Mus Evolution Regulate the Inclusion of Exon 5, which Alters Mouse APOBEC3 Translation Efficiency
Source: PLoS Pathog. 2012 Jan 19;8(1):e1002478. doi: 10.1371/journal.ppat.1002478 (PMC3262013; doi:10.1371/journal.ppat.1002478)
Supplement: Table S2 — Primers used to generate intron 5 deletion mutants and chimeras, for exon 5/intron 5 nucleotide substitutions, and for modification of TCCT repeat and T/C 741 SNP in intron 4. (PDF) [file ppat.1002478.s004.pdf]

Table S2. Primers used to generate intron 5 deletion mutants and chimeras, for exon 5/intron 5 nucleotide substitutions, and for modification of TCCT repeat and T/C 741 SNP in intron 4.

1) Generation of intron 5 deletion mutants from B6 or BALB exon 5-6 plasmid:

A) intron 5-Δ3'
5'-TTACAACCCAGACATCTTTAGGTTC-3'and
5'-CAGTCCCCAAACTTTTCTTATCTT-3'

B) 2000bp intron 5
5'-TTACAACCCAGACATCTTTAGGTTC-3' and
5'-CTTCTGGCCACCTCTGCTCTAC-3'

C) 1100bp intron 5
5'-TTACAACCCAGACATCTTTAGGTTC-3' and
5'-CTGGAGGGCTGGCCAGGTCTGC-3'

D) 600bp intron 5
5'-TTACAACCCAGACATCTTTAGGTTC-3' and
5'-CCAATTGCCAGAGGTACCTTCC-3'

E) 100bp intron 5
5'-TTACAACCCAGACATCTTTAGGTTC-3' and
5'-TACTCTTATCCCTCTCATTCCT-3'

F) 100bp intron 5 (3'-100bp)
5'-AGAAGCCCCTGCATTGGTCCAG-3' and
5'-TACTCTTATCCCTCTCATTCCT-3'.

2) Generation of chimeras between B6 and BALB exon 5-6 plasmids:

G) 5' intron 5 and the vector
5'-CCCTGTTGTTTATTTGCTGTGTGACAGTC-3' and
5'-AACTGCGAGTAAAATTCCTCTTCACTTAGC-3'

H) 3' intron 5 and exon 6 insert:
5'-TTACAACCAACGAGTCAAGCATCTCTGC-3' and
5'-CTGGAGGGCTGGCCAGGTCTGC-3'

3) Site-directed mutagenesis of exon 5/intron 5:

I) BALB C14T
5'-CCTTGCTACATCTCGGTCCCTTCCAGCTCTTCATC-3' and
5'-GATGAAGAGCTGGAAGGGACCGAGATGTAGCAAGG-3'

J) BALB C88G and BALB ΔTCCT C88G
5'-CCAGAGACGAGGTTCTGGGTGGAGGGCAGGTGAGC-3' and
5'-GCTCACCTGCCCTCCACCCAGAACCTCGTCTCTGG-3'

K) BALB C153G G163A
5'-GGCAGGGTACACTTACGTAAGAGGCCAGGGGAGGAGCATTGAGG-3' and
5'-CCTCAATGCTCCTCCCCTGGCCTCTTACGTAAGTGTACCCTGCC-3'

L) B6 T14C and B6 exon 5-6 T14C
5'-CCTTGCTACATCCCGGTCCCTTCCAGCTCTTCATC-3' and
5'-GATGAAGAGCTGGAAGGGACCGGGATGTAGCAAGG-3'

M) B6 G88C, B6 exon 5-6 G88C, B6 +TCCT G88C and B6T14CG88C
5'-CCAGAGACGAGGTTCTGCGTGGAGGGCAGGTGAGC-3' and
5'-CTGCCCTCCACGCAGAACCTCGTCTCTGGG-3'

O) B6 T14C G88C G153C A163G and B6 exon 5-6 T14C G88C G153C A163G

5’-GGCAGGGTACACTTACCTAAGAGGCCGGGGGAGGAGCATTGAGG-3’

and

5’-CCTCAATGCTCCTCCCCCGGCCTCTTAGGTAAGTGTACCCTGCC-3’

P) B6 exon 5-6 G153C

5’-GGGTACACTTACCTAAGAGGCCAGGGGAGGAGC-3’ and
5’-GCTCCTCCCCTGGCCTCTTAGGTAAGTGTACCC-3’

Q) B6 exon 5-6 A163G

5’-GGGTACACTTACGTAAGAGGCCGGGGGAGGAGC-3’ and
5’-GCTCCTCCCCCGGCCTCTTACGTAAGTGTACCC-3’

4) Modifications of intron 4 TCCT repeat and T/C741 SNP:

R) BALB ΔTCCT

5’-CCCCAGGAGAATCCCTGACCATATCCTGGCCCCCTCTTCC-3’ and
5’-AGCAAGGTCTGGAAATGGAAGAGGGGGCCAGGATATGGTCAGGG-3’

S) BALB C741T and BALB ΔTCCT C741T

5’-CCTCACTCTGGTTATCAACACCCCAGGAG-3’ and
5’-CTCCTGGGGTGTTGATAACCAGAGTGAGG-3’

T) B6 +TCCT

5’-CCCCAGGAGAATCCCTGACCATATCCTTCCTGGCCCCCTCTTCC-3’and
5’-GGAAGAGGGGGCCAGGAAGGATATGGTCAGGGATTCTCCTGGGG-3’

U) B6 T741C and B6 +TCCT T741C

5’-CCTCACTCTGGTTACCAACACCCCAGGAG-3’ and
5’-CTCCTGGGGTGTTGGTAACCAGAGTGAGG-3’
